# Exploratory analyses of frequent high-fat food intake in diets and its association with increased odds of atopic dermatitis in Singapore and Malaysia Young Chinese adults

**DOI:** 10.1017/S0007114525000716

**Published:** 2025-04-14

**Authors:** Jun Jie Lim, Kavita Reginald, Yee-How Say, Mei Hui Liu, Fook Tim Chew

**Affiliations:** 1 Department of Biological Sciences, Faculty of Science, National University of Singapore, 117543 Singapore; 2 Department of Biological Sciences, School of Medicine and Life Sciences, Sunway University, Petaling Jaya 47500, Selangor, Malaysia; 3 Department of Biomedical Science, Faculty of Science, Universiti Tunku Abdul Rahman (UTAR), Kampar 31900, Perak, Malaysia; 4 Department of Food Science & Technology, Faculty of Science, National University of Singapore, 117543 Singapore

**Keywords:** Atopic dermatitis, Dietary fats, Dietary habits, Epidemiology, Intake frequencies

## Abstract

High-fat food intake is associated with atopic dermatitis (AD), but the role of habitual dietary habits related to the frequency of high-fat food intake remains unclear. To address this, we developed a frequency-based dietary index, Diet Quality based on Dietary Fat Score, to assess high-fat food intake and examined its association with AD in 13 561 young Chinese adults (mean age = 22·51 years, (sd 5·90)) from Singapore and Malaysia. Using an investigator-administered questionnaire aligned with the validated International Study of Asthma and Allergies in Childhood protocol, we conducted multivariable logistic regression analysis, adjusting for demographics, body mass index, genetic predisposition and lifestyle factors, with false discovery rate correction for multiple comparisons. Frequent high-fat food intake was associated with higher odds of AD (adjusted OR (AOR): 1·53; 95 % CI: 1·31, 1·77; *P*< 0·001). The association remained significant regardless of total fat intake (AOR: 1·45; 95 % CI: 1·05, 1·80; *P*< 0·001) and among individuals with high fruit and vegetable intake (AOR: 1·49; 95 % CI: 1·19, 1·86; *P*< 0·001) or low energy intake (AOR: 1·40; 95 % CI: 1·05, 1·86; *P*< 0·05). No synergistic effects were observed between dietary factors. These findings highlight that frequent intake of high-fat foods is independently associated with AD, emphasising the potential of dietary moderation in AD risk management.

Atopic dermatitis (AD) is a prevalent and complex inflammatory skin disease that can develop in predisposed individuals during adulthood. AD manifests in diverse phenotypes, including disease presentation (clinical manifestation), persistence (symptoms duration), chronicity (long-term progression) and its impact on sleep disturbances (severity)^(1–3)^. These phenotypes are critical for understanding the disease’s varied course, long-term effects and functional impairment. While genetic factors play a key role in AD susceptibility, environmental exposures, lifestyle choices (such as smoking and alcohol consumption) and dietary habits also contribute to the disease’s onset and progression^(3–9)^. Particularly, diet diversification in Asian countries changed dietary habits and food preferences and favoured diets with higher fat content among Asian nations^(10,11)^. While dietary fats are crucial for energy provision and nutrient absorption, excessive intake may contribute to low-grade chronic inflammation, potentially exacerbating allergic diseases such as AD. Most previous research on dietary fats and AD has mainly focussed on paediatric populations, particularly examining the impact of specific essential fatty acids like *n*-3 and *n*-6 long-chain polyunsaturated fatty acids on AD risk and severity. These studies suggest that dietary fats play a role in modulating the inflammatory processes underlying AD^(12)^. However, these studies have largely overlooked broader dietary patterns and the habitual consumption of high-fat foods, especially in transitional age groups like young adults. Young adulthood represents a critical period of dietary shifts and immune system changes. Yet, robust research on the effects of high-fat food consumption on AD in this age group remains limited.

The impact of frequent high-fat food intake, distinct from total fat consumption, on AD development is poorly understood. While total fat intake reflects the overall amount of fat consumed, frequent high-fat food intake emphasises the regularity of consumption, which may influence inflammation and immune function differently. High-fat foods are typically defined based on the percentage of their total calorie content derived from fats. While the specific thresholds can vary, established guidelines often categorised foods as high-fat when they contain more than 17·5 g of total fat per 100 g^(13)^. High-fat foods such as margarine, butter and fast food have been associated with an increased risk of severe eczema in adolescents and children in a global study^(14)^. However, the relationship between high-fat foods and AD is multifaceted, influenced by factors such as family history, lifestyle preferences, overall dietary patterns and other dietary components^(7,12)^.

Given the complexity of dietary fats and their role in AD, this study aims to address a specific gap by focusing on the intake frequency of high-fat food in the young Chinese adult population from Singapore and Malaysia. Using a derived frequency-based dietary index, we explored the association between the frequent consumption of high-fat foods and various AD phenotypes, including symptom presentation, persistency, chronicity and impact on sleep disturbances. This cross-sequential study also examined the specificity of the association by comparing it with other secondary outcomes. Additionally, we assessed the potential differential impact of high-fat food intake *v*. total fat intake on AD presentation.

## Methods

### Study population

Between 2005 and 2022, 18 528 subjects were recruited for the Singapore/Malaysia Cross-sectional Genetics Epidemiology Study^(3,8,9,15–18)^. Recruitment was conducted consecutively from a diverse pool that included university students, university staff and members of the public in Singapore and Malaysia. Efforts were made to minimise selection bias by including all eligible individuals without preference for specific characteristics. This study adheres to the ethical standards of the Declaration of Helsinki and Good Clinical Practices. Approval for the studies conducted at the National University of Singapore (NUS) was granted by the Institutional Review Board (IRB) under reference codes, NUS-07-023, NUS-09-256, NUS-10-445, NUS-13-075, NUS-14-150 and NUS-18-036. In Malaysia, studies conducted at Universiti Tunku Abdul Rahman and Sunway University were approved by Universiti Tunku Abdul Rahman’s Scientific and Ethical Review Committee (Reference code: U/SERC/03/2016) and the Sunway University Research Ethics Committee (Reference code: SUREC 2019/029), respectively. Before participation, all participants provided written informed consent. For participants under 21 years, additional written consent was obtained from a parent or guardian.

Participants of all ethnicities (e.g. Chinese, Malay, Indian and others) were recruited; however, for this study, we focussed on participants of Chinese ethnicity. This decision was made to ensure a sufficiently large and statistically empowered sample size, given that Chinese individuals represent the predominant ethnic group in Singapore (75·2 %)^(19)^. The smaller sample sizes of Malay and Indian participants (*n* < 1000) limited the ability to perform reliable subgroup analyses in these groups. Thus, only the Chinese ethnicity was selected for our final analysis in this study as a starting point to investigate the association between intake frequency of high-fat foods and AD. Future studies will include broader ethnic representation once sufficient sample sizes are available to empower such analyses.

To ensure the robustness of our findings, a separate power calculation was performed following Charan and Biswas^(20)^ to estimate the appropriate sample size for our cross-sequential study, with a primary focus on AD. Assuming a significance level of 0·05 and an estimated diseased prevalence range between 5 % and 20 %, the calculation covers the prevalence rates for other outcomes like asthma and allergic rhinitis (AR)^(21–24)^. Additionally, most of the secondary outcomes in this study, including acne, dry skin and emotional discomfort, have a prevalence rate exceeding 20 %, ensuring that the study is sufficiently powered for all outcomes assessed.

### Atopic dermatitis phenotypes

Information on subjects’ socio-demographic, dietary habits, anthropometry, family atopic history and personal medical history was collected using an investigator-administered questionnaire that followed the standardised International Study of Asthma and Allergies in Childhood protocol^(25)^.

For this study, we followed established guidelines from the Hanifin and Rajka criteria^(26)^ and the UK Working Party’s Diagnostic criteria^(27)^ to classify AD individuals. An individual was considered an ever AD case if they demonstrated a positive response in the skin prick test (SPT) to house dust mite (HDM) allergens (*Blomia tropicalis* and *Dermatophagoides pteronyssinus*) and had a recurrent flexural itchy rash for at least 6 months. These flexural areas include the folds of the elbows, behind the knees, in front of the ankles, under the buttocks or around the neck, cheeks, ears or eyes. Allergic sensitisation was determined by a positive SPT response to HDM allergens, which was used to indicate atopy—a key determinant of AD. Previous allergen studies have supported that IgE sensitisation is highly associated with HDM allergens, with over 80 % of individuals being HDM serum IgE positive^(28–30)^. The SPT protocol was consistent with previous descriptions^(3,8,9,15–18)^. The 6-month period was chosen to differentiate between normal itchy rashes, such as those from insect bites or heat and AD. AD rashes tend to last longer and can be recurrent, with fluctuating flare-ups. This specific combination of 6-month timeframe and flexural distributions ensured that the symptoms were consistent with AD rather than transient skin conditions^(31,32)^. Throughout the collection, our trained personnel concurrently evaluated the presence of a flexural rash on subjects. These evaluations were periodically cross-verified with a dermatologist and found to be concordant. AD presentation was compared between ever AD cases (*n* 2316) and non-allergic non-eczema controls (*n* 3650).

Among individuals with AD presentation (ever AD), we further classified cases based on persistency (recovered AD *v*. current AD), chronicity (acute AD *v*. chronic AD) and severity (mild AD *v*. moderate-severe AD). Those experiencing continuous or intermittent itchy flexural rash throughout the past 12 months were classified as current AD cases. Individuals with a history of AD but who did not experience any itchy flexural rash at any point in the past 12 months were classified as recovered AD. Identifying current AD cases helps predict ongoing challenges by capturing those with recent symptoms and providing insight into the immediate disease burden. For chronicity among ever AD cases, individuals who never achieve complete resolution of their itchy flexural rash within the last 12-month timeframe were classified as chronic AD. Individuals who had AD but achieved full symptom resolution within the past 12 months were identified as acute AD cases. Recognising chronic AD cases provides insights into the long-term nature of the inflammatory skin condition in affected individuals. Finally, various levels of AD severity were determined based on a consistent pattern of experiencing sleep disturbances at night in the last 12 months. Sleep disturbance is a significant indicator of AD severity due to its profound impact on the quality of life^(33)^. The assessment of AD chronicity and AD severity has been extensively validated by International Study of Asthma and Allergies in Childhood^(25)^.

### Secondary outcomes

In addition to investigating the relationship between the frequency of high-fat diet and AD phenotypes, we conducted a multivariable logistic regression analysis to assess various secondary outcomes. These secondary outcomes were selected to ascertain the specificity of the observed association between the frequency of high-fat diet and AD. The included secondary outcomes encompassed other allergic conditions such as HDM allergy (SPT negative individuals *v*. SPT positive individuals), AR (non-allergic non-rhinitis *v*. ever AR cases)^(23)^ and allergic asthma (AS) (non-allergic non-asthma *v*. ever AS cases)^(24)^, as well as acne vulgaris (non-acne *v*. acne cases)^(34)^, dry skin (non-dry skin *v*. dry skin cases), chronic rhinosinusitis (non-chronic rhinosinusitis *v*. chronic rhinosinusitis cases), tooth decay (not having tooth decays *v*. tooth decays cases), hepatitis A virus infection (non-infected *v*. infected cases), emotional discomfort, drug and pain medication allergy (no drug allergy controls *v*. drug allergic cases). The distribution of controls and cases for each outcome was described in online Supplementary Table 1.

### Assessment of dietary habits

A validated semi-quantitative FFQ from the International Study of Asthma and Allergies in Childhood Phase III study was used to examine the dietary habits of various food groups among the subjects^(35)^. There were three available responses: ‘never or only occasionally’, ‘once or twice per week’ and ‘most or all days’. As we were interested in studying the dietary habits of high-fat foods, we stratified the food groups into three fat categories (high-fat, low-fat and negligible dietary fats) based on the information on the average estimated total fat amount (100 g per edible portion) retrieved from the Singapore Health Promotion Board nutritional database^(36)^. To ensure that the categorisation is accurate and representative in reflecting the fat amount in a specified food group, we selected a diverse list of common food items consumed by locals and calculated the average of their estimated fat content (online Supplementary Table 2). This study has lowered the threshold to ≥ 10·0 g of total fat/100 g to capture a broader range of commonly consumed foods that might not be included under the stricter >17·5 g threshold. We also included seafood and milk as high-fat foods despite having an estimated 3–4 g of total fat/100 g. Food groups with high dietary fats were meat, seafood, eggs, milk, margarine, butter and burgers/fast foods, while those with low dietary fats (< 3·0 g of total fat/100 g) were fruit, vegetables, pulses, cereals, rice and potatoes. A separate sensitivity analysis, including those excluding seafood and milk or using different thresholds, demonstrated consistent associations, confirming the robustness of our findings.

To derive Diet Quality based on Dietary Fat Score (DQDFS) index, a specific score was assigned to the corresponding intake frequencies with 0 for never or only occasionally, 2 for once or twice per week and 7 for most or all days. This is consistent with the rubrics established^(37)^. A positive score was prepended to high-fat food groups while a negative score was prepended to low-fat food groups. The summation of these assigned dietary fat scores (DFS) for all thirteen food groups resulted in DQDFS (online Supplementary Figure 1). In this study, cut-offs for DQDFS were set at the 33rd and 66th percentiles based on a preliminary analysis of the Singapore/Malaysia Cross-sectional Genetics Epidemiology Study cohort (*n* 13 561) (online Supplementary Figure 2). This ensured balanced and representative categorisation into low (DFS ≤ –8), moderate (DFS between –7 and –1) and high (DFS ≥ 0) while maintaining sufficient statistical power. Sensitivity analyses using alternative thresholds (e.g. median split and 25th/75th percentiles) confirmed the robustness of these cut-offs, as the strength and direction of the association between DQDFS and AD remained consistent across all categorisations (online Supplementary Table 3). In a previous study, we developed an amount-based dietary index, Diet Quality based on Total Fat Amount (DQTFA), to evaluate subjects’ dietary fat intake^(38)^. Combining DQTFA and DQDFS indices enables a more thorough assessment of individuals’ dietary habits related to high-fat foods, encompassing both the frequency and quantity of consumption.

### Covariates

Numerical variables like the Asian class body mass index (BMI), age and categorical variables like sex, parental eczema, alcohol intake and smoking status were identified as potential confounders and rigorously adjusted for in multivariable analyses^(3–9)^. Energy intake was analysed in stratified models as a positive covariate, while fruit and vegetable intake was included as a negative covariate^(39)^. Total energy intake (KJ/serving/week [1 kcal = 4.184 KJ]) was categorised into low, moderate or high intake using the 33rd (29,059 kJ/serving/week [6943 kcal]) and 66th (43,162 kJ/serving/week [10,312 kcal]) percentiles and derived from the same Health Promotion Board nutritional database. This approach ensured a robust adjustment to isolate the independent effect of dietary fat intake on AD.

### Statistical analysis

Logistic regression analysis was used to determine the associations between various AD phenotypes and dietary indices. The results were presented in adjusted odds ratio (AOR), 95 % confidence intervals (CI) and *P*-value. A chi-square analysis was used to determine the presence of a significant difference in the subject distribution for a given categorical variable between the DFS categories. A synergy factor (SF) analysis was conducted following the approach described by Cortina-Borja *et al.* ^(40)^ to assess potential synergistic interactions between various dietary factors, specifically the frequency of high-fat food intake, fruit and vegetable intake and total energy intake, in influencing AD susceptibility. SF analysis is useful for identifying interactions that may not be evident when dietary components are studied individually. A synergistic effect occurs when the combined effect of two dietary factors is greater than the sum of their individual effects, while an antagonistic effect arises when the combined effect is less than the sum of individual effects. The SF value quantifies these interactions, with values greater than 1 indicating synergism, and values lesser than 1 indicating antagonism. To account for multiple comparisons and minimise the risk of type I errors, *P*-values obtained from all analyses were adjusted using the false discovery rate method^(41)^. This adjustment ensures the statistical reliability of our findings while maintaining control over the proportion of false positives in exploratory analyses. All data used in the analysis were processed using Microsoft Excel (http://office.microsoft.com/en-us/excel/) with statistical analysis conducted in R statistical language (RStudio Team Version 2021.09.0.351, 2021).

## Results

### Dietary fat scores among Singapore and Malaysia Chinese population

Approximately one-third of the 13 561 subjects in the Singapore/Malaysia Cross-sectional Genetics Epidemiology Study cohort fell into each DFS category. The majority of subjects had a high DFS (34·3%) followed by those with a low DFS (32·0 %) and a moderate DFS (29·4%) as detailed in Table 1. Due to some invalid or missing responses regarding dietary habits for certain food groups, 576 subjects (4·25%) could not be accurately categorised into any of the DFS categories and were thus excluded in the subsequent analysis.

**Table 1.** An overview of the demographic distribution of 13 561 young Chinese adults from the Singapore/Malaysia Cross-sectional Genetics Epidemiology Study (SMCGES) cohort. Participants were categorised based on their dietary fat scores using the frequency-based dietary index, Diet Quality based on Dietary Fat Score (DQDFS)

Variables	Diet Quality based on Dietary Fat Score (DQDFS)^*^								Chi-square *P*-value^**†**^		
	Low DFS		Moderate DFS		High DFS		Overall population		Low DFS *v*. Moderate DFS	Moderate DFS *v*. High DFS	Low DFS *v*. High DFS
	(*n* 4343)		(*n* 3984)		(*n* 4658)		(*n* 13 561)				
	Mean	sd	Mean	sd	Mean	sd	Mean	sd			
Age	22·91	6·58	22·52	5·89	22·11	5·09	22·51	5·90	–	–	–
	*n*	%	*n*	%	*n*	%	*n*	%			
Sex											
Female	2829	65·1 %	2345	58·9 %	2526	54·2 %	8060	59·4 %	**< 0·001**	**< 0·001**	**< 0·001**
Male	1514	34·9 %	1639	41·1 %	2132	45·8 %	5501	40·6 %			
BMI, Asian class (kg/m^2^) (*n*, %)											
Healthy (18·0–23·0)	2402	55·3 %	2223	55·8 %	2519	54·1 %	7445	54·9 %	0·15	0·12	**0·001**
Underweight (< 18·0)	785	18·1 %	668	16·8 %	770	16·5 %	2327	17·2 %			
Overweight (> 23·0)	615	14·2 %	607	15·2 %	781	16·8 %	2092	15·4 %			
NA	541	12·5 %	486	12·2 %	588	12·6 %	1697		–	–	–
Parental eczema (*n*, %)											
No	3744	86·2 %	3507	88·0 %	4082	87·6 %	11 831	87·2 %	**< 0·05**	0·34	0·14
Yes	538	12·4 %	429	10·8 %	533	11·4 %	1558	11·5 %			
NA	61	1·4 %	48	1·2 %	43	0·9 %	172		–	–	–
Tobacco smoking status (*n*, %)											
Non-smoker	3982	91·7 %	3663	91·9 %	4258	91·4 %	12 395	91·4 %	**< 0·05**	**< 0·05**	**< 0·001**
Ex-smoker	23	0·5 %	42	1·1 %	63	1·4 %	135	1·00 %			
Smoker	50	1·2 %	44	1·1 %	81	1·7 %	183	1·35 %			
NA	288	6·6 %	235	5·9 %	256	5·5 %	848		–	–	–
Use of alcohol (*n*, %)											
Non-drinker	2114	48·7 %	1688	42·4 %	2499	53·6 %	5830	43·0 %	**< 0·001**	**< 0·001**	**< 0·0001**
Occasional Drinker	1897	43·7 %	1990	49·9 %	1785	38·3 %	6643	49·0 %			
Frequent Drinker	58	1·3 %	77	1·9 %	132	2·8 %	277	2·04 %			
NA	274	6·3 %	229	5·7 %	242	5·2 %	811		–	–	–

DFS, Dietary fat score.

* A total of 576 participants were excluded because they did not fit into any of the DQDFS categories.

† A chi-square *P*-value < 0·05 was adjusted by false discovery rate for multiple comparisons. Statistically significant *P*-values were bolded.

Our population has a mean age of 22·51 (sd 5·90)). Over half of the subjects were females, with the largest proportion of females (male: female ratio; 1:1·87) having a low DFS. The difference in sex proportion across DFS categories was significant. More than half of the individuals had a healthy BMI range across the DFS categories. There was a pronounced difference between the proportion of overweight individuals in the low DFS (14·2 %) and high DFS (16·8 %) categories, suggesting that more individuals with an overweight BMI frequently adhered to a diet consisting of more high-fat foods. In contrast, the proportion of underweight individuals decreased with increasing DFS. The observation was not coincidental with a previous study reported that individuals with higher BMI have a greater tendency to seek higher energy-dense foods^(42)^. The distribution between individuals across DFS showed significant differences in tobacco smoking and alcohol consumption while there were significant differences between those with low DFS and moderate DFS.

### Frequent high-fat diet intake and its association with various outcomes

There were significant differences in the distribution of cases and controls across DFS categories for AD, AR, AS and HDM allergy, with a gradually increasing proportion of diseased individuals observed in higher DFS categories (online Supplementary Table 4). A high DFS was associated with higher odds for certain allergic and inflammatory conditions such as AD, AR, AS, HDM allergy and dry skin (*P*< 0·05). However, after correction for multiple comparisons, only the association with AD (AOR: 1·55; 95 % CI: 1·34, 1·80; *P*< 0·001), AR (AOR: 1·26; 95 % CI: 1·09, 1·44; adjusted *P*< 0·01), AS (AOR: 1·28; 95 % CI: 1·09, 1·50; *P*< 0·05) and HDM allergy (AOR: 1·38; 95 % CI: 1·25, 1·52; *P*< 0·001) remained significant. A risk relationship was observed with increased intake of high-fat foods across all allergic diseases (AD, AR, AS), with the association being most pronounced for AD. No significant associations were found for other secondary outcomes included in the analysis (Table 2).

**Table 2.** Association between the intake frequency of high-fat foods, as assessed by the frequency-based dietary index – Diet Quality based on Dietary Fat Score (DQDFS) and various outcomes among 13 561 young Chinese adults from the Singapore/Malaysia Cross-sectional Genetics Epidemiology Study (SMCGES) cohort

Outcome	AOR	95 % CI	*P*
I. Ever atopic dermatitis (AD) (non-allergic non-eczema *v*. AD cases)			
Low dietary fat score	1·00	REF	–
Moderate dietary fat score	1·25	1·07, 1·45	**0·01**
High dietary fat score	1·55	1·34, 1·80	**< 0·001**
II. Ever allergic rhinitis (AR) (non-allergic non-rhinitis *v*. AR cases)			
Low dietary fat score	1·00	REF	–
Moderate dietary fat score	1·21	1·05, 1·40	**0·01**
High dietary fat score	1·26	1·09, 1·44	**<0·01**
III. Ever allergic asthma (AS) (non-allergic non-asthma *v*. AS cases)			
Low dietary fat score	1·00	REF	–
Moderate dietary fat score	1·19	1·01, 1·40	0·04
High dietary fat score	1·28	1·09, 1·50	**<0·01**
IV. House dust mites (HDM) allergy (skin prick test (SPT) negative individuals *v*. SPT positive individuals)			
Low dietary fat score	1·00	REF	–
Moderate dietary fat score	1·18	1·06, 1·30	**<0·01**
High dietary fat score	1·38	1·25, 1·52	**< 0·001**
V. Acne vulgaris (non-acne *v*. acne cases)			
Low dietary fat score	1·00	REF	–
Moderate dietary fat score	1·08	0·95, 1·24	0·25
High dietary fat score	0·98	0·96, 1·11	0·73
VI. Dry skin (non-dry skin *v*. dry skin cases)			
Low dietary fat score	1·00	REF	–
Moderate dietary fat score	1·21	1·02, 1·43	0·03
High dietary fat score	1·17	1·00, 1·37	0·05
VII. Chronic rhinosinusitis (non-chronic rhinosinusitis *v*. chronic rhinosinusitis cases)			
Low dietary fat score	1·00	REF	–
Moderate dietary fat score	1·14	0·92, 1·42	0·24
High dietary fat score	1·07	0·87, 1·32	0·52
VIII. Tooth decay (no tooth decay *v*. tooth decay)			
Low dietary fat score	1·00	REF	–
Moderate dietary fat score	1·09	0·81, 1·48	0·56
High dietary fat score	0·89	0·67, 1·19	0·43
IX. Hepatitis A virus (HAV) infection (non-infected *v*. infected cases)			
Low dietary fat score	1·000	REF	–
Moderate dietary fat score	0·67	0·14, 2·73	0·58
High dietary fat score	1·02	0·28, 3·73	0·98
X. Emotional discomfort (control *v*. cases)			
Low dietary fat score	1·00	REF	–
Moderate dietary fat score	1·01	0·74, 1·39	0·94
High dietary fat score	1·06	0·78, 1·43	0·72
XI. Drug allergy (no drug allergy *v*. drug allergy)			
Low dietary fat score	1·00	REF	–
Moderate dietary fat score	0·99	0·86, 1·14	0·84
High dietary fat score	0·92	0·80, 1·06	0·24
XII. Pain medication allergy (no pain drug allergy *v*. pain drug allergy)			
Low dietary fat score	1·00	REF	–
Moderate dietary fat score	1·30	0·72, 2·35	0·38
High Dietary Fat Score	1·12	0·63, 2·03	0·70

Results are from multivariable logistic regression analysis on the variables associated with various outcomes as indicated. Values are presented as adjusted odds ratio (AOR), 95 % confidence intervals (CI) and *P*-value. *P*-value was adjusted by false discovery rate for multiple comparisons, and *P*-value < 0·05 was statistically significant and written in bold. *P*-values > 0·05 were not statistically significant. Multivariable analysis was adjusted for age, sex, body mass index (Asian class), parental eczema, tobacco smoking and use of alcohol.

### Association between frequent high-fat diet intake and atopic dermatitis

**Figure 1.**
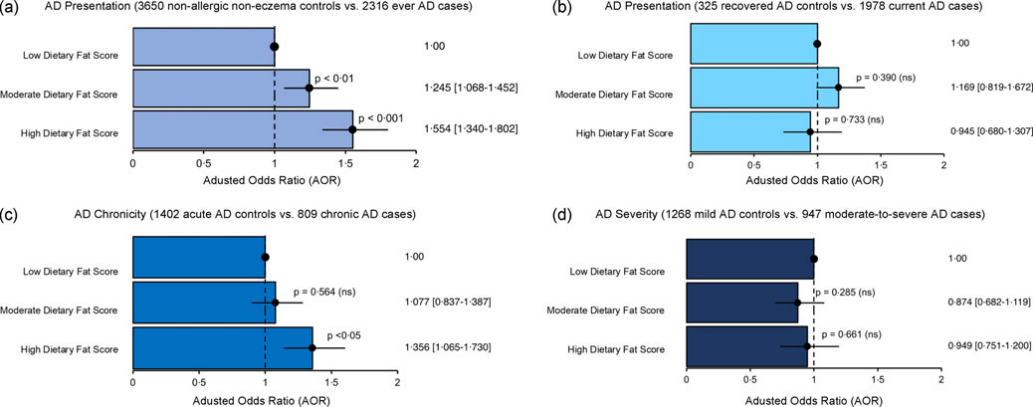
OR plot illustrating the association between the frequency-based dietary index (Diet Quality based on Dietary Fat Score (DQDFS) and various atopic dermatitis (AD) phenotypes, (a) AD presentation, (b) AD persistency, (c) AD chronicity and (d) AD severity. Results are presented in adjusted odds ratios (AOR), 95 % confidence intervals (CI) and *P*-value. *P*-value was adjusted by false discovery rate for multiple comparisons and *P*-value < 0·05 to be statistically significant and written in bold. Multivariable analysis was adjusted for age, sex, body mass index (Asian class), parental eczema, tobacco smoking and use of alcohol. A reference dotted line is drawn at the interception point where AOR equals 1·00, and the 95 % CI is represented by a single line that cuts the AOR.

Among the various AD phenotypes, a high DFS was significantly associated with higher odds for AD presentation and AD chronicity (AOR: 1·36; 95 % CI: 1·07, 1·73; *P*< 0·05). In both associations, there was a gradual increase in the AOR from moderate DFS to high DFS, indicating higher associated odds with frequent intake of high-fat foods in diets (Figure 1). However, AD persistency and severity were not significantly associated with DFS.

**Figure 2.**
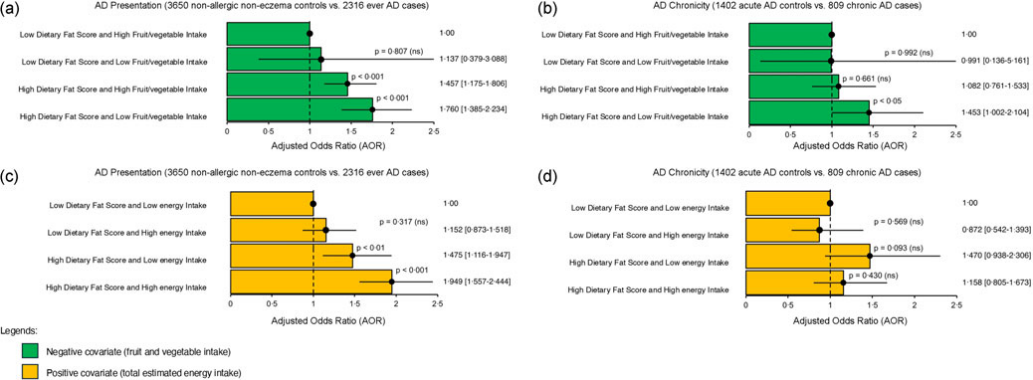
OR plot illustrating the association between the frequency-based dietary index (Diet Quality based on Dietary Fat Score (DQDFS) and AD phenotypes, stratified by covariates: negative (fruit and vegetable intake) and positive (energy intake). Results are presented in adjusted odds ratio (AOR), 95 % confidence intervals (CI) and *P*-value. *P*-value was adjusted by false discovery rate for multiple comparisons and *P*-value < 0·05 to be statistically significant and written in bold. Multivariable analysis was adjusted for age, sex, body mass index (Asian class), parental eczema, tobacco smoking and use of alcohol. A reference dotted line is drawn at the interception point where AOR equals 1·00 and the 95 % CI is represented by a single line that cuts the AOR.

Subsequently, we further reanalysed the association between AD presentation and AD chronicity with high-fat diet intake by stratifying according to fruit and vegetable intake (as a negative covariate) and energy intake (as a positive covariate) separately. This approach further assesses the independence of the observed association from other dietary factors, elucidating potential confounding effects and identifying effect modifiers. The effect of high DFS on AD presentation was not confounded by high fruit and vegetable intake (AOR: 1·46; 95 % CI: 1·18, 1·81; *P*< 0·001) (Figure 2(a)) and energy intake (AOR: 1·48; 95 % CI: 1·12, 1·95; *P*< 0·01) (Figure 2(c)). Additionally, there was a gradual increment in AOR associated with high DFS as fruit and vegetable intake decreased and energy intake increased. Although SF analysis indicated the absence of a synergistic interaction between the dietary factors examined (online Supplementary Table 5 (I) and (II)), they might act independently to influence AD presentation. In contrast, the association between DFS and AD chronicity was not statistically significant in high fruit and vegetable intake (Figure 2(b)) or low energy intake (Figure 2(d)).

### Association between frequency and dietary fat intake with atopic dermatitis

**Figure 3.**
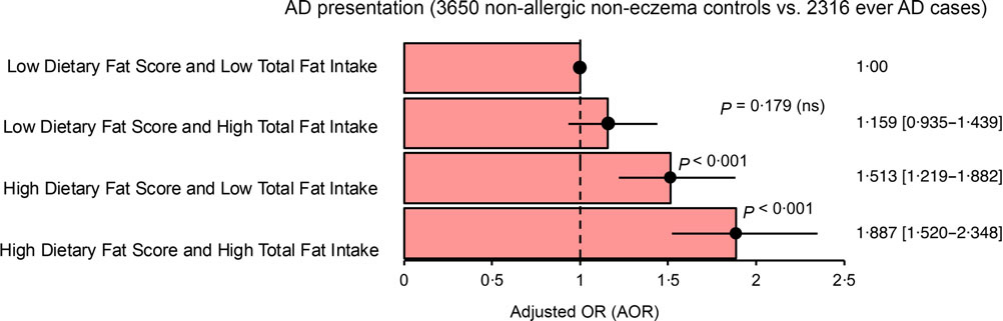
OR plot illustrating the association between combined dietary indices (Diet Quality based on Dietary Fat Score (DQDFS) and Diet Quality based on Total Fat Amount (DQTFA)) and AD presentation. DQDFS assesses intake frequency of high-fat foods, while DQTFA assesses total estimated dietary fat intake. Results are presented in adjusted odds ratios (AOR), 95 % confidence intervals (CI) and *P*-value. *P*-value < 0·05 to be statistically significant. Multivariable analysis was adjusted for age, sex, body mass index (Asian class), parental eczema, tobacco smoking and use of alcohol. A reference dotted line is drawn at the interception point where AOR equals 1·00, and the 95 % CI is represented by a single line that cuts the AOR.

Finally, we studied the association between dietary habits characterised by frequent intake of high-fat food diets and high total dietary fat intake with the presentation of AD. Understanding this distinction is crucial, as it provides insights into how different aspects of dietary fat consumption may impact the manifestation of AD. Compared with individuals who infrequently consumed high-fat foods while maintaining a low dietary fat intake, those with increased frequency of high-fat food intake exhibited higher associated odds for AD presentation (AOR: 1·51; 95 % CI: 1·22, 1·88; *P*< 0·001). Similarly, the associated odds for AD presentation increased further with higher dietary fat intake (AOR: 1·89; 95 % CI: 1·52, 2·35; *P*< 0·001) (Figure 3). Notably, the influence of high-fat food intake frequency on AD odds was independent of the overall intake amount (online Supplementary Table 5 (III)). Taken together, the key findings emphasised the importance of moderating the intake frequency of high-fat foods as potentially more crucial for lowering the odds associated with AD presentation than solely focusing on reducing overall dietary fat intake.

## Discussion

Our cross-sequential study found a significant association between frequent high-fat food intake and the presentation AD, but not its severity or persistency. The association was specific to AD and did not extend to other non-allergic related secondary outcomes, such as acne, drug allergies, tooth decay, dry skin, hepatitis A virus infection or emotional discomfort. Additionally, there was a similar risk relationship between high-fat food intake and other allergic conditions, including HDM allergy, AS and AR. Interestingly, more frequent consumption of high-fat foods, even in lower amounts, was associated with higher odds of AD presentation. These findings suggest that the frequency of high-fat food consumption, rather than solely the overall amount of dietary fat, may play a critical role in AD susceptibility, offering a potential target for dietary interventions aimed at reducing the occurrence of AD.

High-fat diets are implicated in promoting AD and allergic diseases through several common underlying mechanisms. First, excessive dietary fat can dysregulate macrophage function, increasing inflammation^(43)^. Second, lipid mediators, particularly saturated fatty acids like palmitic acid, activate immune cells via toll-like receptor (TLR) 2 and TLR4, triggering inflammatory responses^(44–46)^. Third, high-fat intake can disrupt gut microbiota, compromising gut integrity and increasing inflammatory cell infiltration^(47)^. Our findings support an association between high-fat food intake and AD, particularly in symptom presentation and chronicity. However, the absence of significant associations with AD severity and persistency suggests distinct underlying mechanisms. AD severity was defined in our study by late-night sleep disturbances due to itching and itch-associated neuromodulators and cytokines like interleukin-31 and thymic stromal lymphopoietin and it may not be influenced by high-fat intake^(48,49)^. For AD persistency, the small number of recovered AD cases may have reduced statistical power, making associations harder to detect in this study. Additionally, confounding factors such as antioxidant-rich diets or poor sleep habits could influence inflammatory pathways, modulating symptoms persistence^(50,51)^.

To better understand the complex role of dietary fats in the severity and persistency of AD, future studies should integrate functional assessments of microbiome composition, immune responses and metabolomic and lipidomic profiles, alongside evaluations of sleep patterns and concurrent dietary habits. These investigations can be achieved through multi-omics approaches, incorporating stool and blood analyses to capture metabolic and immunological markers, actigraphy to monitor sleep disturbances and detailed dietary logs or 24-hour recalls to examine overall nutrition^(52)^. Combining these methods will provide a holistic view of how dietary fats, lifestyle factors and individual metabolic variations influence long-term AD progression and symptom persistence.

The retrospective nature of our study limits our ability to establish a causal relationship between the frequency of high-fat food intake and AD phenotypes. While our findings suggest an association between frequent intake of high-fat foods and AD presentation, caution is warranted in interpreting these findings. Dietary habits alone are neither exclusive nor sufficient to entirely modify AD risk, as other factors, such as sleep patterns and allergen exposures, also contribute to AD susceptibility^(51,53)^. Furthermore, individual differences, cultural influences and personal eating experiences can shape dietary habits and their impact on AD progression and management^(54)^. Therefore, the study’s findings should be interpreted in consideration of these potential confounding factors and future research would benefit from a more comprehensive assessment of such variables. Randomised controlled trials focusing on dietary interventions to reduce the frequency of high-fat food intake are needed to clarify the temporal sequence between dietary fats and AD. We also acknowledge the potential for recall or reporting bias in dietary data. To address this, we implemented several measures to mitigate these limitations. The direct administration of a validated FFQ by trained investigators provided participants with the opportunity to clarify any doubts during data collection. The FFQ was designed to be simple, straightforward and cost-effective for assessing dietary habits in a large and diverse population. Moreover, the FFQ was validated for the adult population^(55)^ with the protocol has been adjusted to include dietary questions applicable to adults, with guidelines for customising the food list to regional dietary patterns^(56)^. Lastly, our study highlights the need for broader ethnic representation. Future studies should aim to replicate these findings in other ethnic groups, including Malay, Indian and other Asian populations, to enhance the generalisability of the results. To achieve this, we plan to leverage large, independent datasets in Singapore, such as the multiethnic cohort population^(57)^, the Growing Up in Singapore Towards Healthy Outcomes longitudinal birth cohort^(58)^ and the Health for Life in Singapore (HELIOS) population cohort^(59)^. Additionally, we propose conducting randomised controlled trials to further validate the observed associations and establish causal relationships. Such efforts will provide deeper insights into how dietary habits and cultural factors uniquely influence AD across diverse ethnicities.

In conclusion, our study highlights an association between the frequent intake of high-fat foods and AD. These findings emphasise the clinical importance of moderation in dietary fat intake as a potential and promising strategy for managing AD. While further research is necessary to explore underlying mechanisms and validate these results across diverse populations, our study provides a strong foundation for investigating dietary interventions aimed at reducing high-fat food intake to mitigate AD symptoms and improve AD outcomes.

## Supporting information

Lim et al. supplementary material 1 Lim et al. supplementary material

Lim et al. supplementary material 2 Lim et al. supplementary material

Lim et al. supplementary material 3 Lim et al. supplementary material

Lim et al. supplementary material 4 Lim et al. supplementary material

Lim et al. supplementary material 5 Lim et al. supplementary material

Lim et al. supplementary material 6 Lim et al. supplementary material

Lim et al. supplementary material 7 Lim et al. supplementary material

Lim et al. supplementary material 8 Lim et al. supplementary material

## Supplementary material

For supplementary material accompanying this paper visit https://doi.org/10.1017/S0007114525000716
