# Supplementary material for: Exploratory analyses of frequent high-fat food intake in diets and its association with increased odds of atopic dermatitis in Singapore and Malaysia Young Chinese adults
Source: Br J Nutr. 2025 Apr 4;133(7):977–86. doi: 10.1017/S0007114525000716 (PMC12198345; doi:10.1017/S0007114525000716)
Supplement: Lim et al. supplementary material 1 — Lim et al. supplementary material [file S0007114525000716sup001.zip › bjn_2500071_ES/Editing and TypeSetting PO_bjn_2500071.pdf]

Cambridge Journals

1 April 2[illegible]

Purchase order for copy editing and setting Open Acce[illegible]

Please refer to the proofing instructions for this journal
Proofs MUST be delivered by the time agreed in the service level agreement

article no. BJN-2024-0777 setters no. 2500071 bjn 0 running order

first named author Jun Jie Lim author email: dbscft@nus.edu.sg

corresp au Fook Tim CHEW

title Exploratory Analyses of Frequent High-Fat Food Intake in Diets and Its Association with Increased Odds of Atop[illegible] Dermatitis in Singapore and Malaysia Young Chinese Adults

doi 10.1017/S0007114525000716 no. of book revs open_access_article [illegible]

submission dates received 18/7/24 revised 10/2/25 accepted 21/3/25

article type **Research Article**

print colour **yes** web colour **no** Figures 1, 2, 3

copyright line © The Author(s), [YYYY]. Published by Cambridge University Press on behalf of The Nutrition Societ[illegible] This is an Open Access article, distributed under the terms of the Creative Commons Attribution licen[illegible] (http://creativecommons.org/licenses/by/4.0), which permits unrestricted re- use, distribution and reproduction, provided the original article is properly cited.

notes for setter Please note and tag with subject category

supplementary materials Yes Supplemental Tables 1 and 2. Supplemental Figures 1-4

Cambridge Journals

1 April 2(

Purchase order for copy editing and setting Open Acces

funding body Singapore Immunology Network

author country

assoc ed
