# Supplementary material for: Exploratory analyses of frequent high-fat food intake in diets and its association with increased odds of atopic dermatitis in Singapore and Malaysia Young Chinese adults
Source: Br J Nutr. 2025 Apr 4;133(7):977–86. doi: 10.1017/S0007114525000716 (PMC12198345; doi:10.1017/S0007114525000716)
Supplement: Lim et al. supplementary material 2 — Lim et al. supplementary material [file S0007114525000716sup002.docx]

**Supplemental Figure 1.** Flowchart illustrating the design of the frequency-based dietary index, Diet Quality based on Dietary Fat Score (DQDFS).
