# Supplementary material for: Exploratory analyses of frequent high-fat food intake in diets and its association with increased odds of atopic dermatitis in Singapore and Malaysia Young Chinese adults
Source: Br J Nutr. 2025 Apr 4;133(7):977–86. doi: 10.1017/S0007114525000716 (PMC12198345; doi:10.1017/S0007114525000716)
Supplement: Lim et al. supplementary material 3 — Lim et al. supplementary material [file S0007114525000716sup003.docx]

**Supplemental Figure 2.** The distribution of dietary fat scores (DFS) among young Chinese adults from the Singapore/Malaysia Cross-sectional Genetics Epidemiology Study (SMCGES) cohort. The cut-offs, as represented by the dotted lines, are selected at the 33^rd^ and 66^th^ percentile based on the preliminary population analysis to categorize subjects.
