# Supplementary material for: Exploratory analyses of frequent high-fat food intake in diets and its association with increased odds of atopic dermatitis in Singapore and Malaysia Young Chinese adults
Source: Br J Nutr. 2025 Apr 4;133(7):977–86. doi: 10.1017/S0007114525000716 (PMC12198345; doi:10.1017/S0007114525000716)
Supplement: Lim et al. supplementary material 4 — Lim et al. supplementary material [file S0007114525000716sup004.docx]

**Supplemental Table 1.** Distribution of individuals from the Singapore/Malaysia Cross-sectional Genetics Epidemiology Study (SMCGES) cohort for various outcomes.

| Outcomes | **Controls** | **Cases** | **NA** |
| --- | --- | --- | --- |
| **Atopic Dermatitis (AD) Presentation**  (3650 non-allergic non-eczema controls vs. 2316 ever AD cases) | 3650 (26.9%) | 2316 (17.1%) | 7595 (56.0%) |
| **AD Persistency**  (325 recovered AD controls vs. 1979 current AD cases) | 325 (2.4%) | 1979 (14.6%) | 11257 (83.0%) |
| **AD Chronicity**  (1402 acute AD controls vs. 809 chronic AD cases) | 1402 (10.3%) | 809 (6.0%) | 11350 (83.7%) |
| **AD Severity**  (1268 mild AD controls vs. 947 moderate-to-severe AD cases) | 1268 (9.4%) | 947 (7.0%) | 11346 (83.7%) |
| **Allergic Rhinitis (AR) Presentation**  (2133 non-allergic non-rhinitis controls vs. 4327 ever AR cases) | 2133 (15.7%) | 4237 (31.2%) | 7107 (52.4%) |
| **Allergic Asthma (AS) Presentation**  (3466 non-allergic non-asthmatic controls vs. 2010 ever AS cases) | 3466 (25.6%) | 2010 (14.8%) | 8085 (59.6%) |
| **House Dust Mites (HDM) Allergy**  (4622 non-HDM allergic controls vs. 8840 HDM allergic cases) | 4622 (34.1%) | 8840 (65.2%) | 99 (0.7%) |
| **Acne Vulgaris**  (3228 non-acne controls vs. 3764 acne cases) | 3228 (23.8%) | 3764 (27.8%) | 6569 (48.4%) |
| **Dry Skin**  (2492 non-dry skin controls vs. 2110 dry skin cases) | 2492 (18.4%) | 2110 (15.6%) | 8959 (66.1%) |
| **Chronic Rhinosinusitis**  (1504 non-chronic rhinosinusitis controls vs. 1375 chronic rhinosinusitis cases) | 1504 (11.1%) | 1375 (10.1%) | 10682 (78.8%) |
| **Tooth Decay**  (620 not having tooth decays vs. 632 tooth decays cases) | 620 (4.6%) | 632 (4.7%) | 12309 (90.8%) |
| **Hepatitis A Virus (HAV) Infection**  (non-infected controls vs. Infected cases) | 12299 (90.7%) | 21 (0.2%) | 1241 (9.2%) |
| **Emotional Discomfort**  (747 controls vs. 513 cases) | 747 (5.5%) | 513 (3.8%) | 12031 (88.7%) |
| **Drug Allergy**  (11,388 non-drug allergic controls vs. 2173 drug allergic cases) | 11388 (84.0%) | 2173 (16.0%) | 0 (0.0%) |
| **Pain Medication Allergy**  (6468 controls vs. 88 cases) | 6468 (47.7%) | 88 (0.6%) | 7005 (51.7%) |
