# Supplementary material for: Exploratory analyses of frequent high-fat food intake in diets and its association with increased odds of atopic dermatitis in Singapore and Malaysia Young Chinese adults
Source: Br J Nutr. 2025 Apr 4;133(7):977–86. doi: 10.1017/S0007114525000716 (PMC12198345; doi:10.1017/S0007114525000716)
Supplement: Lim et al. supplementary material 5 — Lim et al. supplementary material [file S0007114525000716sup005.docx]

**Supplemental Table 2.** Estimated total fat amount, based on the usual serving size, retrieved from the Health Promotion Board, Singapore, nutritional database. **(a)** The average estimated total fat amount (grams per 100g edible portion) for each food type and used as a reference for the categorization of high and low dietary fat foods in Diet Quality based on Dietary Fat Score (DQDFS). **(b)** List of specific foods adopted for the averaging of estimated total fat amount for each food type.

| 1. **Food Types** | **Grams/100g Edible Portion** |
| --- | --- |
| **High-fat Foods^1^** | |
| - Butter | 82.00 |
| - Margarine | 64.00 |
| - Burgers/fast food | 15.00 |
| - Eggs | 13.00 |
| - Meat (e.g., Beef, lamb, chicken, pork) | 13.00 |
| **Low-fat Foods^2^** | |
| - Seafood (including fish) | 3.80 |
| - Milk | 3.32 |
| - Cereals (including bread) | 2.10 |
| - Rice | 0.50 |
| - Fruits | 0.20 |
| - Potatoes | 0.20 |
| - Pulses (peas, beans, lentils) | 0.20 |
| - Vegetables (green and root) | 0.20 |
| Information on estimated total fat amount (grams per 100g edible portion) was retrieved on the Health Promotion Board, Singapore nutritional database at <https://focos.hpb.gov.sg/eservices/ENCF/>. The data was accessed in June 2023. The average estimated total fat amount was calculated based on the list of common foods under each food type and values are rounded off to the nearest 2 decimal place. ^1^The general guideline for high total fat in foods ≥ 17.5g/100g while low total fat in foods **≤** 3g/100g, but for the interest of this study, we chose a stricter threshold as we only account for limited food groups in our questionnaire. | |

| 1. **List of Common Foods Adopted from Health Promotion Board, Singapore** | **Grams/100g Edible Portion** | |  |
| --- | --- | --- | --- |
| **Butter** | | |  |
| - Butter, unsalted | 83.20 | |  |
| - Butter, salted | 80.80 | |  |
| **Margarine** | | |  |
| - Margarine spread, monounsaturated | 69.60 | |  |
| - Margarine spread, polyunsaturated | 69.50 | |  |
| - Margarine spread, polyunsaturated, reduced fat, reduced salt | 60.00 | |  |
| - Margarine spread, monounsaturated, reduced fat, reduced salt | 56.40 | |  |
| **Burgers/fast food** | | |  |
| - KFC cheese fries | 22.31 | |  |
| - Onion rings, small, Burger King | 18.69 | |  |
| - Winglets, KFC | 17.95 | |  |
| - Drumstick, original recipe, KFC | 17.08 | |  |
| - Apple pie, McDonald's | 15.29 | |  |
| - McSpicy, McDonald's | 12.68 | |  |
| - Hamburger, Burger King | 12.17 | |  |
| - Filet-o-fish, McDonald's | 11.03 | |  |
| - McChicken, McDonald's | 10.17 | |  |
| - Hawaiian 12" Large Pan Pizza, Pizzahut | 9.40 | |  |
| **Eggs** | | |  |
| - Egg, goose, whole, raw | 15.60 | |  |
| - Egg, duck, whole, raw | 13.00 | |  |
| - Egg, hen, whole, raw | 11.10 | |  |
| **Meat (e.g., Beef, lamb, chicken, pork)** | | |  |
| - Pork, boneless, unspecified cut, cooked, lean and fat | 22.00 | |  |
| - Mutton cooked | 13.80 | |  |
| - Beef, boneless, unspecified cut, raw, lean and fat | 11.70 | |  |
| - Chicken, broilers or fryers, drumstick, meat and skin, cooked, stewed) | 10.64 | |  |
| - Beef, boneless, unspecified cut, cooked, lean only) | 6.30 | |  |
| **Milk** | | |  |
| - Milk, whole, evaporated, canned | 8.10 | |  |
| - Milk, whole | 3.70 | |  |
| - Full cream milk | 3.40 | |  |
| - UHT chocolate milk | 1.90 | |  |
| - Soya bean milk, with sugar (hawker centre) | 1.60 | |  |
| - Low fat milk | 1.20 | |  |
| **Seafood (including fish)** | | | |
| - Molluscs, scallop, mixed species, cooked, breaded and fried | | 10.94 | |
| - Steamed fish, unspecified | | 3.33 | |
| - Crab, freshwater, boiled | | 2.30 | |
| - Cuttlefish, cooked | | 1.40 | |
| - Prawn, king, cooked | | 0.90 | |
| **Cereals (including cereals)** | | | |
| - Breakfast cereal, plain, contains whole grains | | 3.10 | |
| - Bread, brown | | 2.50 | |
| - Bread, French | | 1.80 | |
| - Bread, white | | 1.00 | |
| **Rice** | | | |
| - Brown rice cooked | | 0.80 | |
| - White rice cooked | | 0.50 | |
| - Rice, glutinous, white, cooked | | 0.19 | |
| **Potatoes** | | | |
| - Sweet potato, boiled, without skin | | 0.30 | |
| - Potato, old, baked, flesh and skin | | 0.10 | |
| **Pulses (peas, beans, lentils)** | | | |
| - Snow peas, raw | | 0.30 | |
| - Beans, rench, raw | | 0.20 | |
| - Longbeans, boiled | | 0.10 | |
| **Vegetables (green and root)** | | | |
| - Vegetable, dark green non-leafy, unspecified, boiled | | 0.30 | |
| - Vegetable, dark green leafy, unspecified, boiled, drained | | 0.25 | |
| - Carrot, boiled | | 0.18 | |
| - Onion, ginger, garlic raw | | 0.00 | |
| **Fruits** | | | |
| - Banana, raw, unspecified type | | 0.33 | |
| - Watermelon | | 0.30 | |
| - Apples, raw, without skin | | 0.13 | |
| - Orange | | 0.10 | |
| - Papaya | | 0.10 | |
| - Pear, green, raw with peel | | 0.00 | |
| Information on estimated total fat amount (grams per 100g edible portion) was retrieved on the Health Promotion Board, (HPB) Singapore nutritional database at <https://focos.hpb.gov.sg/eservices/ENCF/>. The data was accessed in June 2023. This list of foods was selected to represent some of the more commonly consumed foods by local Singaporeans. A diversity of food is considered in the calculation of the average estimated total fat amount for each food type to ensure the best representation. The estimated total fat amount value of each food is rounded off to the nearest 2 decimal place. | | | |
