# Supplementary material for: Exploratory analyses of frequent high-fat food intake in diets and its association with increased odds of atopic dermatitis in Singapore and Malaysia Young Chinese adults
Source: Br J Nutr. 2025 Apr 4;133(7):977–86. doi: 10.1017/S0007114525000716 (PMC12198345; doi:10.1017/S0007114525000716)
Supplement: Lim et al. supplementary material 6 — Lim et al. supplementary material [file S0007114525000716sup006.docx]

**Supplemental Table 3.** Sensitivity analysis of the association between DQDFS and AD using different cut-off thresholds.

| Sensitivity Analysis | Using 33^rd^ and 66^th^ percentiles | Using 25^th^ and 75^th^ percentiles | Using 50^th^ (median) percentile |
| --- | --- | --- | --- |
| **Distribution of Participants (*n*, %)** | | | |
| Low DFS | 4343 (32.0%) | 3343 (24.7%) | 6248 (46.1%) |
| Moderate DFS | 3984 (29.4%) | 5953 (43.9%) | - |
| High DFS | 4658 (34.3%) | 3689 (27.2%) | 6737 (49.7%) |
| NA | 576 (4.25%) | | |
| **Cut-off Scores** | | | |
| Low DFS | ≦ -8 | ≦ -10 | <3 |
| Moderate DFS | Between -7 and -1 | Between -9 and 1 | - |
| High DFS | ≧ 0 | 2 | ≧ -4 |

| Sensitivity Analysis | Using 33^rd^ and 66^th^ percentiles | | | Using 25^th^ and 75^th^ percentiles | | | Using 50 percentile | | |
| --- | --- | --- | --- | --- | --- | --- | --- | --- | --- |
|  | OR | 95% CI | P | OR | 95% CI | P | OR | 95% CI | P |
| Low DFS | 1.000 | REF | - | 1.000 | REF | - | 1.000 | REF | - |
| Moderate  DFS | 1.236 | 1.082-1.413 | 0.002 (**) | 1.171 | 1.027-1.336 | 0.019 (*) | - | - | - |
| High DFS | 1.624 | 1.429-1.848 | <0.001 (***) | 1.742 | 1.507-2.014 | <0.001 (***) | 1.453 | 1.306-1.617 | <0.001 (***) |

P-value was adjusted by False Discovery Rate (FDR) for multiple comparisons and p-value < 0.05 was statistically significant and written in bold. P-values > 0.05 was not statistically significant (ns).
