# Supplementary material for: Exploratory analyses of frequent high-fat food intake in diets and its association with increased odds of atopic dermatitis in Singapore and Malaysia Young Chinese adults
Source: Br J Nutr. 2025 Apr 4;133(7):977–86. doi: 10.1017/S0007114525000716 (PMC12198345; doi:10.1017/S0007114525000716)
Supplement: Lim et al. supplementary material 7 — Lim et al. supplementary material [file S0007114525000716sup007.docx]

**Supplemental Table 4.** Distribution of individuals from the Singapore/Malaysia Cross-sectional Genetics Epidemiology Study (SMCGES) cohort across different outcomes based on dietary fat scores.

| **Outcomes** | **Low Dietary Fat Score**  **(*n* = 4343)** | **Moderate Dietary Fat Score**  **(*n* = 3984)** | **High Dietary Fat Score**  **(*n* = 4658)** | **Chi-square p-value^1^** |
| --- | --- | --- | --- | --- |
| **Primary Outcome** | | | | |
| 1. **Atopic Dermatitis (AD) Presentation** | | | | |
| Non-allergic non-eczema controls | 1328 (30.6%) | 1079 (27.1%) | 1073 (23.0%) | **<0.001 (***)** |
| Ever AD cases | 672 (15.5%) | 675 (16.9%) | 882 (18.9%) |  |
| **Secondary Outcomes** | | | | |
| 1. **Allergic Rhinitis (AR) Presentation** | | | | |
| Non-allergic non-rhinitis controls | 761 (17.5%) | 602 (15.1%) | 669 (14.4%) | **<0.001 (***)** |
| Ever AR cases | 1297 (29.9%) | 1274 (32.0%) | 1595 (34.2%) |  |
| 1. **Allergic Asthma (AS) Presentation** | | | | |
| Non-allergic non-asthmatic controls | 1255 (28.9%) | 1013 (25.4%) | 1043 (22.4%) | **<0.001 (***)** |
| Ever AS cases | 613 (14.1%) | 601 (15.1%) | 725 (15.6%) |  |
| 1. **House Dust Mites (HDM) Allergy** | | | | |
| Non-HDM allergic controls | 1675 (38.6%) | 1358 (34.1%) | 1376 (29.5%) | **<0.001 (***)** |
| HDM allergic cases | 2638 (60.7%) | 2593 (65.1%) | 3257 (69.9%) |  |
| 1. **Acne Vulgaris** | | | | |
| Non-acne controls | 974 (22.4%) | 926 (23.2%) | 1191 (25.6%) | 0.247 (ns) |
| Acne cases | 1105 (25.4%) | 1155 (29.0%) | 1368 (29.4%) |  |
| 1. **Dry Skin** | | | | |
| Non-dry skin controls | 760 (17.5%) | 698 (17.5%) | 929 (19.9%) | 0.09367 (ns) |
| Dry skin cases | 592 (13.6%) | 642 (16.1%) | 804 (17.3%) |  |
| 1. **Chronic Rhinosinusitis** | | | | |
| Non-chronic rhinosinusitis controls | 450 (10.4%) | 439 (11.0%) | 573 (12.3%) | 0.1165 (ns) |
| Chronic rhinosinusitis cases | 369 (8.5%) | 436 (10.9%) | 546 (11.7%) |  |
| 1. **Tooth Decay** | | | | |
| Not having tooth decays | 164 (3.8%) | 183 (4.6%) | 258 (5.5%) | 0.1969 (ns) |
| Tooth decay cases | 181 (4.2%) | 207 (5.2%) | 234 (5.0%) |  |
| 1. **Hepatitis A Virus (HAV) Infection** | | | | |
| Non-infected controls | 3999 (92.1%) | 3634 (91.2%) | 4205 (90.3%) | 0.3688 (ns) |
| Infected cases | 8 (0.2%) | 3 (0.1%) | 8 (0.2%) |  |
| 1. **Emotional Discomfort** | | | | |
| Controls | 212 (4.9%) | 236 (5.9%) | 287 (6.2%) | 0.6279 (ns) |
| Cases | 133 (3.1%) | 156 (3.9%) | 206 (4.4%) |  |
| 1. **Drug Allergy** | | | | |
| Non-drug allergic controls | 3601 (82.9%) | 3352 (84.1%) | 3972 (85.3%) | 0.009 (**) |
| Drug allergic Cases | 742 (17.1%) | 632 (15.9%) | 686 (14.7%) |  |
| 1. **Pain Medication Allergy** | | | | |
| Controls | 2095 (48.2%) | 1846 (46.3%) | 2286 (49.1%) | 0.7836 (ns) |
| Cases | 26 (0.6%) | 26 (0.7%) | 34 (0.7%) |  |

^1^Chi-square p-value was adjusted by False Discovery Rate (FDR) for multiple comparisons and p-value < 0.05 was statistically significant and written in bold. P-values > 0.05 was not statistically significant (ns).
