# Supplementary material for: Exploratory analyses of frequent high-fat food intake in diets and its association with increased odds of atopic dermatitis in Singapore and Malaysia Young Chinese adults
Source: Br J Nutr. 2025 Apr 4;133(7):977–86. doi: 10.1017/S0007114525000716 (PMC12198345; doi:10.1017/S0007114525000716)
Supplement: Lim et al. supplementary material 8 — Lim et al. supplementary material [file S0007114525000716sup008.docx]

**Supplemental Table 5.** Synergy factor (SF) analysis for the interactions between dietary fat score (DFS) and various dietary factors in influencing the associated risks for atopic dermatitis (AD) presentation and chronicity, among 13,561 young Chinese adults from the Singapore/Malaysia Cross-sectional Genetics Epidemiology Study (SMCGES) cohort.

| 1. **Interactions Between Dietary Fat Scores (DFS) and Fruit/vegetable Intake** | | | | | | | |
| --- | --- | --- | --- | --- | --- | --- | --- |
| - **AD Presentation (3650 non-allergic non-eczema controls vs. 2316 ever AD cases)** | | | | | | | |
| DFS | Fruit/vegetable Intake | Non-Allergic Non-Eczema Controls | AD Cases | Odds Ratio | SF P Value | SF  (95% CI) | Interaction |
| Low | High | 1034 | 511 | Reference | 0.695 | 0.828  (0.321-2.133) | NA |
| Low | Low | 11 | 8 | 1.472 |  |  |  |
| High | High | 379 | 289 | 1.543 |  |  |  |
| High | Low | 253 | 235 | 1.880 |  |  |  |
| - **AD Chronicity (1402 acute AD controls vs. 809 chronic AD cases)** | | | | | | | |
| DFS | Fruit/vegetable Intake | Acute AD Controls | Chronic  AD Cases | Odds Ratio | SF P Value | SF  (95% CI) | Interaction |
| Low | High | 309 | 182 | Reference | 0.282 | 2.477 (0.475-12.917) | NA |
| Low | Low | 6 | 2 | 0.566 |  |  |  |
| High | High | 178 | 94 | 0.897 |  |  |  |
| High | Low | 127 | 94 | 1.257 |  |  |  |
| 1. **Interactions Between Dietary Fat Scores (DFS) and Energy Intake** | | | | | | | |
| - **AD Presentation (3650 non-allergic non-eczema controls vs. 2316 ever AD cases)** | | | | | | | |
| DFS | Energy Intake | Non-Allergic Non-Eczema Controls | AD Cases | Odds Ratio | SF P Value | SF  (95% CI) | Interaction |
| Low | Low | 575 | 258 | Reference | 0.786 | 1.048  (0.749-1.465) | NA |
| Low | High | 300 | 165 | 1.226 |  |  |  |
| High | Low | 247 | 172 | 1.552 |  |  |  |
| High | High | 482 | 431 | 1.993 |  |  |  |
| - **AD Chronicity (1402 acute AD controls vs. 809 chronic AD cases)** | | | | | | | |
| DFS | Energy Intake | Acute AD Controls | Chronic  AD Cases | Odds Ratio | SF P Value | SF  (95% CI) | Interaction |
| Low | Low | 157 | 95 | Reference | 0.521 | 0.834  (0.479-1.452) | NA |
| Low | High | 101 | 53 | 0.867 |  |  |  |
| High | Low | 93 | 75 | 1.333 |  |  |  |
| High | High | 259 | 151 | 0.964 |  |  |  |
| 1. **Interactions Between Dietary Fat Score (DFS) and Total Estimated Dietary Fat Intake Amount** | | | | | | | |
| 1. **AD Presentation (3650 non-allergic non-eczema controls vs. 2316 ever AD cases)** | | | | | | | |
| DFS | Fat Intake | Non-Allergic Non-Eczema Controls | AD Cases | Odds Ratio | SF P Value | SF  (95% CI) | Interaction |
| Low | Low | 625 | 282 | Reference | 0.787 | 0.965 (0.745-1.250) | NA |
| Low | High | 703 | 390 | 1.230 |  |  |  |
| High | Low | 558 | 421 | 1.672 |  |  |  |
| High | High | 515 | 461 | 1.984 |  |  |  |
| 1. **AD Chronicity (1402 acute AD controls vs. 809 chronic AD cases)** | | | | | | | |
| DFS | Fat Intake | Acute AD Controls | Chronic  AD Cases | Odds Ratio | SF P Value | SF  (95% CI) | Interaction |
| Low | Low | 168 | 106 | Reference | 0.868 | 0.964  (0.629-1.479) | NA |
| Low | High | 244 | 129 | 0.838 |  |  |  |
| High | Low | 237 | 164 | 1.097 |  |  |  |
| High | High | 279 | 156 | 0.886 |  |  |  |
