# Supplementary material for: Exploratory analyses of frequent high-fat food intake in diets and its association with increased odds of atopic dermatitis in Singapore and Malaysia Young Chinese adults
Source: Br J Nutr. 2025 Apr 4;133(7):977–86. doi: 10.1017/S0007114525000716 (PMC12198345; doi:10.1017/S0007114525000716)
Supplement: Lim et al. supplementary material 1 — Lim et al. supplementary material [file S0007114525000716sup001.zip › bjn_2500071_ES/bjn_2500071_ES/bjn-2024-0777-20250328160540.zip!/bjn-2024-0777-20250328160540/doc/Cleaned Manuscript (AD & Fats).docx]

# 1 Title Page

## 1.1 Article Title

Exploratory Analyses of Frequent High-Fat Food Intake in Diets and Its Association with Increased Odds of Atopic Dermatitis in Singapore and Malaysia Young Chinese Adults

## 1.2 Short Title

Dietary Habits and Atopic Dermatitis

## 1.3 Authors Information

Jun Jie LIM, BSc(Hons)^1^: lim.jun.jie@u.nus.edu

Kavita REGINALD, Ph.D *^1,2^ :kavitar@sunway.edu.my

Yee-How SAY, Ph.D *^1,2,3^ :yeehows@sunway.edu.my

Mei Hui LIU, Ph.D ^4^: fstlmh@nus.edu.sg

Fook Tim CHEW, Ph.D **^1^ : dbscft@nus.edu.sg

## 1.4 Authors Affiliations

^1^Department of Biological Sciences, Faculty of Science, National University of Singapore, Singapore 117543.

^2^Department of Biological Sciences, School of Medicine and Life Sciences, Sunway University, 47500 Petaling Jaya, Selangor, Malaysia.

^3^Department of Biomedical Science, Faculty of Science, Universiti Tunku Abdul Rahman (UTAR), 31900 Kampar, Perak, Malaysia.

^4^Department of Food Science & Technology, Faculty of Science, National University of Singapore, Singapore 117543.

*Current Address: ^2^Department of Biological Sciences, School of Medicine and Life Sciences, Sunway University, 47500 Petaling Jaya, Selangor, Malaysia.

## 1.5 Corresponding Author

**Corresponding Author:

CHEW Fook Tim, PhD

Associate Professor, Department of Biological Sciences; Vice Dean, Faculty of Science National University of Singapore, Allergy and Molecular Immunology Laboratory, Lee Hiok Kwee Functional Genomics Laboratories, Block S2, Level 5, 14 Science Drive 4, off Lower Kent Ridge Road, Singapore 117543.

Phone: +65 65161685; Fax: +65 67792486

Email: dbscft@nus.edu.sg

## 1.6 Abbreviations

AD: Atopic dermatitis; AOR: Adjusted odds ratio; AR: Allergic rhinitis; AS: Allergic asthma; BMI: Body mass index; CI: Confidence intervals; DQDFS: Dietary quality based on dietary fat score; DFS: Dietary fat score; DQTFA: Diet quality based on total fat amount; FFQ: Food frequency questionnaire; HAV: Hepatitis A virus; HBP: Health promotion board; HDM: House dust mites; IgE: Immunoglobulin E; ISAAC: International study of asthma and allergies in childhood; SF: Synergy factor; SMCGES: Singapore/Malaysia cross-sectional genetics epidemiology study; SPT: Skin prick test; TLR: Toll-like receptor.

# 2 Abstract

High-fat food intake is associated with atopic dermatitis (AD), but the role of habitual dietary habits related to the frequency of high-fat food intake remains unclear. To address this, we developed a frequency-based dietary index, Diet Quality based on Dietary Fat Score, to assess high-fat food intake and examined its association with AD in 13,561 young Chinese adults (mean age = 22.51 years, SD ± 5.90) from Singapore and Malaysia. Using an investigator-administered questionnaire aligned with the validated International Study of Asthma and Allergy in Childhood protocol, we conducted multivariable logistic regression adjusting for demographics, body mass index, genetic predisposition, and lifestyle factors, with false discovery rate correction for multiple comparisons. Frequent high-fat food intake was associated with higher odds of AD presentation (Adjusted Odds Ratios [AOR]: 1.525; 95% Confidence Intervals [CI]: 1.314-1.772; adjusted p < 0.001). The association remained significant regardless of total fat intake (AOR: 1.445; 95% CI: 1.054-1.801; adjusted p < 0.001) and among individuals with high fruit and vegetable intake (Adjusted Odds Ratios [AOR]: 1.489; 95% Confidence Intervals [CI]: 1.191-1.860; adjusted p < 0.001) or low energy intake (AOR: 1.399; 95% CI: 1.054-1.857; adjusted p < 0.05). No synergistic effects were observed between dietary factors. These findings highlight that frequent intake of high-fat foods is independently associated with AD, emphasizing the importance of dietary moderation in AD risk management.

**Keywords**: Atopic Dermatitis, Dietary Fats, Dietary Habits, Epidemiology, Intake Frequencies

# 3 Introduction

Atopic dermatitis (AD) is a prevalent and complex inflammatory skin disease that can develop in predisposed individuals during adulthood. AD manifests in diverse phenotypes, including disease presentation (clinical manifestation), persistence (symptoms duration), chronicity (long-term progression), and its impact on sleep disturbances (severity) [1-3]. These phenotypes are critical for understanding the disease’s varied course, long-term effects, and functional impairment. While genetic factors play a key role in AD susceptibility, environmental exposures, lifestyle choices (such as smoking and alcohol consumption), and dietary habits also contribute to the disease’s onset and progression [3-9]. Particularly, diet diversification in Asian countries changed dietary habits and food preferences and favoured diets with higher fat content among Asian nations [10, 11]. While dietary fats are crucial for energy provision and nutrient absorption, excessive intake may contribute to low-grade chronic inflammation, potentially exacerbating allergic diseases such as AD. Most previous research on dietary fats and AD has mainly focused on paediatric populations, particularly examining the impact of specific essential fatty acids like n-3 and n-6 long-chain polyunsaturated fatty acids on AD risk and severity. These studies suggest that dietary fats play a role in modulating the inflammatory processes underlying AD [12]. However, these studies have largely overlooked broader dietary patterns and the habitual consumption of high-fat foods, especially in transitional age groups like young adults. Young adulthood represents a critical period of dietary shifts and immune system changes. Yet, robust research on the effects of high-fat food consumption on AD in this age group remains limited.

The impact of frequent high-fat food intake, distinct from total fat consumption, on AD development is poorly understood. While total fat intake reflects the overall amount of fat consumed, frequent high-fat food intake emphasizes the regularity of consumption, which may influence inflammation and immune function differently. High-fat foods are typically defined based on the percentage of their total calorie content derived from fats. While the specific thresholds can vary, established guidelines often categorized foods as high-fat when they contain more than 17.5g of total fat per 100g [13]. High-fat foods such as margarine, butter, and fast food have been associated with an increased risk of severe eczema in adolescents and children in a global study [14]. However, the relationship between high-fat foods and AD is multifaceted, influenced by factors such as family history, lifestyle preferences, overall dietary patterns, and other dietary components [7, 12].

Given the complexity of dietary fats and their role in AD, this study aims to address a specific gap by focusing on the intake frequency of high-fat food in the young Chinese adult population from Singapore and Malaysia. Using a derived frequency-based dietary index, we explored the association between frequent consumption of high-fat foods and various AD phenotypes, including symptom presentation, persistency, chronicity, and impact on sleep disturbances. This cross-sequential study also examined the specificity of the association by comparing it with other secondary outcomes. Additionally, we assessed the potential differential impact of high-fat food intake versus total fat intake on AD presentation.

# 4 Methods

## 4.1 Study Population

Between 2005 and 2022, 18,528 subjects were recruited for the Singapore/Malaysia Cross-sectional Genetics Epidemiology Study (SMCGES) [3, 8, 9, 15-18]. Recruitment was conducted consecutively from a diverse pool that included university students, university staff, and members of the public in Singapore and Malaysia. Efforts were made to minimize selection bias by including all eligible individuals without preference for specific characteristics. This study adheres to the ethical standards of the Declaration of Helsinki and Good Clinical Practices. Approval for the studies conducted at the National University of Singapore (NUS) was granted by the Institutional Review Board (IRB) under reference codes, NUS-07-023, NUS-09-256, NUS-10-445, NUS-13-075, NUS-14-150, and NUS-18-036. In Malaysia, studies conducted at Universiti Tunku Abdul Rahman (UTAR) and Sunway University were approved by UTAR’s Scientific and Ethical Review Committee (Reference code: U/SERC/03/2016) and the Sunway University Research Ethics Committee (Reference code: SUREC 2019/029), respectively. Before participation, all participants provided written informed consent. For participants under 21 years, additional written consent was obtained from a parent or guardian.

Participants of all ethnicities (e.g., Chinese, Malay, Indian, and others) were recruited; however, for this study, we focused on participants of Chinese ethnicity. This decision was made to ensure a sufficiently large and statistically empowered sample size, given that Chinese individuals represent the predominant ethnic group in Singapore (75.2%) [19]. The smaller sample sizes of Malay and Indian participants (*n* < 1000) limited the ability to perform reliable subgroup analyses in these groups. Thus, only the Chinese ethnicity was selected for our final analysis in this study as a starting point to investigate the association between intake frequency of high-fat foods and AD. Future studies will include broader ethnic representation once sufficient sample sizes are available to empower such analyses.

To ensure the robustness of our findings, a separate power calculation was performed following Charan and Biswas [20] to estimate the appropriate sample size for our cross-sequential study, with a primary focus on AD. Assuming a significance level of 0.05 and an estimated diseased prevalence range between 5% and 20%, the calculation covers the prevalence rates for other outcomes like asthma and allergic rhinitis [21-24]. Additionally, most of the secondary outcomes in this study, including acne, dry skin, and emotional discomfort, have a prevalence rate exceeding 20%, ensuring that the study is sufficiently powered for all outcomes assessed.

## 4.2 Atopic Dermatitis (AD) Phenotypes

Information on subjects’ sociodemographic, dietary habits, anthropometry, and familial/personal medical history was collected using an investigator-administered questionnaire that followed the standardized International Study of Asthma and Allergy in Childhood (ISAAC) protocol [25].

For this study, we followed established guidelines from the Hanifin and Rajka criteria [26] and the United Kingdom Working Party’s Diagnostic criteria [27] to classify AD individuals. An individual was considered an ever AD case if they demonstrated a positive response in the skin prick test (SPT) to house dust mite allergens (HDM) (*Blomia tropicalis* and *Dermatophagoides pteronyssinus*) and had a recurrent flexural itchy rash for at least six months. These flexural areas include the folds of the elbows, behind the knees, in front of the ankles, under the buttocks, or around the neck, cheeks, ears or eyes. Allergic sensitization was determined by a positive SPT response to HDM allergens, which was used to indicate atopy—a key determinant of AD. Previous allergen studies have supported that immunoglobulin E (IgE) sensitization is highly associated with HDM allergens, with over 80% of individuals being HDM serum IgE positive [28-30]. The SPT protocol was consistent with previous descriptions [3, 8, 9, 15-18]. The 6-month period was chosen to differentiate between normal itchy rashes, such as those from insect bites or heat, and AD. AD rashes tend to last longer and can be recurrent, with fluctuating flare-ups. This specific combination of 6-month timeframe and flexural distributions ensured that the symptoms were consistent with AD rather than transient skin conditions [31, 32]. Throughout the collection, our trained personnel concurrently evaluated the presence of a flexural rash on subjects. These evaluations were periodically cross-verified with a dermatologist and found to be concordant. AD presentation was compared between ever AD cases (*n* = 2316) and non-allergic non-eczema controls (*n* = 3650).

Among individuals with AD presentation (ever AD), we further classified cases based on persistency (recovered AD vs. current AD), chronicity (acute AD vs. chronic AD), and severity (mild AD vs. moderate-severe AD). Those experiencing continuous or intermittent itchy flexural rash throughout the past 12 months were classified as current AD cases. Individuals with a history of AD but did not experience any itchy flexural rash at any point in the past 12 months were classified as recovered AD. Identifying current AD cases helps predict ongoing challenges by capturing those with recent symptoms and providing insight into the immediate disease burden. For chronicity among ever AD cases, individuals who never achieve complete resolution of their itchy flexural rash within the last 12-month timeframe were classified as chronic AD. Individuals who had AD but achieved full symptom resolution within the past 12 months were identified as acute AD cases. Recognizing chronic AD cases provides insights into the long-term nature of the inflammatory skin condition in affected individuals. Finally, various levels of AD severity were determined based on a consistent pattern of experiencing sleep disturbances at night in the last 12 months. Sleep disturbance is a significant indicator of AD severity due to its profound impact on the quality of life [33]. The assessment of AD chronicity and AD severity has been extensively validated by ISAAC [25].

## 4.3 Secondary Outcomes

In addition to investigating the relationship between the frequency of high-fat diets and AD phenotypes, we conducted a multivariable logistic regression analysis to assess various secondary outcomes. These secondary outcomes were selected to ascertain the specificity of the observed association between the frequency of high-fat diets and AD. The included secondary outcomes encompassed other allergic conditions such as HDM allergy (SPT negative individuals vs SPT positive individuals), allergic rhinitis (AR) (non-allergic non-rhinitis vs ever AR cases) [23] and allergic asthma (AS) (non-allergic non-asthma vs ever AS cases) [24], as well as acne vulgaris (non-acne vs acne cases) [34], dry skin (non-dry skin vs dry skin cases), chronic rhinosinusitis (non-chronic rhinosinusitis vs chronic rhinosinusitis cases), tooth decay (not having tooth decays vs tooth decays cases), hepatitis A virus (HAV) infection (non-infected vs infected cases), emotional discomfort, drug and pain medication allergy (no drug allergy controls vs drug allergic cases). The distribution of controls and cases for each outcome was described in **Supplemental Table 1.**

## 4.4 Assessment of Dietary Habits

A validated semi-quantitative food frequency questionnaire (FFQ) from the ISAAC Phase III study was used to examine the dietary habits of various food groups among the subjects [35]. There were three available responses: “never or only occasionally”, “once or twice per week”, and “most or all days”. As we are interested in studying the dietary habits of high-fat foods, we stratified the food groups into three fat categories (high-fat, low-fat, and negligible dietary fats) based on the information on the average estimated total fat amount (100g per edible portion) retrieved from the Singapore Health Promotion Board (HPB) nutritional database [36]. To ensure that the categorization is accurate and representative in reflecting the fat amount in a specified food group, we selected a diverse list of common food items consumed by locals and calculated the average of their estimated fat content (**Supplemental Table 2**). This study has lowered the threshold to ≥10.0g of total fat/100g to capture a broader range of commonly consumed foods that might not be included under the stricter >17.5g threshold. We also included seafood and milk as high-fat foods despite having an estimated 3-4g of total fat/100g. Food groups with high dietary fats were meat, seafood, eggs, milk, margarine, butter, and burgers/fast foods, while those with low dietary fats (<3.0g of total fat/100g) were fruit, vegetables, pulses, cereals, rice, and potatoes. A separate sensitivity analysis, including those excluding seafood and milk or using different thresholds, demonstrated consistent associations, confirming the robustness of our findings.

To derive Diet Quality based on Dietary Fat Score (DQDFS) index, a specific score was assigned to the corresponding intake frequencies with 0 for never or only occasionally, 2 for once or twice per week, and 7 for most or all days. This is consistent with the rubrics established [37]. A positive score was prepended to high-fat food groups while a negative score was prepended to low-fat food groups. The summation of these assigned dietary fat scores (DFS) for all 13 food groups resulted in DQDFS (**Supplemental Figure 1**). In this study, cut-offs for DQDFS were set at the 33^rd^ and 66^th^ percentiles based on a preliminary analysis of the SMCGES cohort (*n* = 13,561) (**Supplemental Figure 2**). This ensured balanced and representative categorization into low (DFS ≤ -8), moderate (DFS between -7 and -1), and high (DFS ≥ 0) while maintaining sufficient statistical power. Sensitivity analyses using alternative thresholds (e.g., median split and 25^th^/75^th^ percentiles) confirmed the robustness of these cut-offs, as the strength and direction of the association between DQDFS and AD remained consistent across all categorizations (**Supplemental Table 3**). In a previous study, we developed an amount-based dietary index, Diet Quality based on Total Fat Amount (DQTFA), to evaluate subjects’ dietary fat intake [38]. Combining DQTFA and DQDFS enables a more thorough assessment of individuals’ dietary habits related to high-fat foods, encompassing both the frequency and quantity of consumption.

## 4.5 Covariates

Numerical variables like the Asian class body mass index (BMI), age and categorical variables like sex, parental eczema, alcohol intake, and smoking status were identified as potential confounders and rigorously adjusted for in multivariable analyses [3-9]. Energy intake was analysed in stratified models as a positive covariate, while fruit and vegetable intake was included as a negative covariate [39]. Total energy intake (kcal/serving/week) was categorized into low, moderate, or high intake using the 33^rd^ (6943 kcal/serving/week) and 66^th^ (10,312 kcal/serving/week) percentiles and derived from the same HPB nutritional database. This approach ensured robust adjustment to isolate the independent effect of dietary fat intake on AD.

## 4.6 Statistical Analysis

All data used in the analysis were processed using Microsoft Excel (http://office.microsoft.com/en-us/excel/) with statistical analysis conducted in R statistical language (RStudio Team Version 2021.09.0.351, 2021). Logistic regression analysis was used to determine the associations between various AD phenotypes and dietary indices. The results were presented in adjusted odds ratio (AOR), 95% confidence intervals (CI), and p-value. A chi-square analysis was used to determine the presence of a significant difference in the subject distribution for a given categorical variable between the DFS categories. A synergy factor (SF) analysis was conducted following the approach described by Cortina-Borja *et al*. [40] to assess potential synergistic interactions between various dietary factors, specifically the frequency of high-fat food intake, fruit and vegetable intake, and total energy intake, in influencing AD susceptibility. SF analysis is useful for identifying interactions that may not be evident when dietary components are studied individually. A synergistic effect occurs when the combined effect of two dietary factors is greater than the sum of their individual effects, while an antagonistic effect arises when the combined effect is less than the sum of individual effects. The SF value quantifies these interactions, with values greater than 1 indicating synergism, and values lesser than 1 indicating antagonism. To account for multiple comparisons and minimize the risk of type I errors, p-values obtained from all analyses were adjusted using the False Discovery Rate (FDR) method [41]. This adjustment ensures the statistical reliability of our findings while maintaining control over the proportion of false positives in exploratory analyses.

# 5 Results

## 5.1 Dietary Fat Scores Among Singapore and Malaysia Chinese Population

Approximately one-third of the 13,561 subjects in the SMCGES cohort fell into each DFS category. The majority of subjects had a high DFS (34.3%), followed by those with a low DFS (32.0 %) and a moderate DFS (29.4%) as detailed in **Table 1**. Due to some invalid or missing responses regarding dietary habits for certain food groups, 576 subjects (4.25%) could not be accurately categorized into any of the DFS categories and were thus excluded in the subsequent analysis.

Our population has a mean age of 22.51 (SD ± 5.90). Over half of the subjects were females, with the largest proportion of females (male: female ratio; 1:1.87) having a low DFS. The difference in sex proportion across DFS categories was significant. More than half of the individuals have a healthy BMI range across the DFS categories. There was a pronounced difference between the proportion of overweight individuals in the low DFS (14.2%) and high DFS (16.8%) categories, suggesting that more individuals with an overweight BMI frequently adhered to a diet consisting of more high-fat foods. In contrast, the proportion of underweight individuals decreased with increasing DFS. The observation was not coincidental with a previous study reported that individuals with higher BMI have a greater tendency to seek higher energy-dense foods [42]. The distribution between individuals across DFS showed some significant differences in tobacco smoking and alcohol consumption while there are significant differences between those with low DFS and moderate DFS.

## 5.2 Frequent High-Fat Diet Intake and Its Association with Various Outcomes

There were significant differences in the distribution of cases and controls across DFS categories for AD, AR, AS, and HDM allergy, with a gradually increasing proportion of diseased individuals observed in higher DFS categories (**Supplemental Table 4**). A high DFS was associated with higher odds for certain allergic and inflammatory conditions such as AD, AR, AS, HDM allergy, and dry skin (p < 0.05), suggesting a potential common shared pathogenesis involving inflammation and immune dysregulation with increased intake of high-fat foods in diets. However, after correction for multiple comparisons, only the association with AD (AOR: 1.554; 95% CI: 1.340-1.802; adjusted p < 0.001), AR (AOR: 1.255; 95% CI: 1.092-1.442; adjusted p < 0.01), AS (AOR: 1.279; 95% CI: 1.089-1.503; adjusted p < 0.05), and HDM allergy (AOR: 1.376; 95% CI: 1.246-1.519; adjusted p < 0.001) remained significant. A dose-response relationship was observed with increased intake of high-fat foods across all allergic diseases (AD, AR, AS), with the association being most pronounced for AD. No significant associations were found for other secondary outcomes included in the analysis (**Table 2**).

## 5.3 Association Between Frequent High-Fat Diet Intake and AD

Among the various AD phenotypes, a high DFS was significantly associated with higher odds for AD presentation and AD chronicity (AOR: 1.356; 95% CI: 1.065-1.730; adjusted p < 0.05). In both associations, there was a dose-dependent increase in the AOR from moderate DFS to high DFS, indicating higher associated odds with frequent intake of high-fat foods in diets (**Figure 1**). However, AD persistency and severity were not significantly associated with DFS. This suggests that while consuming high-fat foods more frequently has a more pronounced associated odds with the development of AD and its chronicity, it may not differentiate between individuals with present or severity of symptoms.

Subsequently, we conducted a re-analysis to investigate the association between AD presentation and AD chronicity with high-fat diet intake by stratifying according to fruit and vegetable intake (as a negative covariate) and energy intake (as a positive covariate) separately. This approach further assesses the independence of the observed association from other dietary factors, elucidating potential confounding effects and identifying effect modifiers. The effect of high DFS on AD presentation was not confounded by high fruit and vegetable intake (AOR: 1.457; 95% CI: 1.175-1.806; adjusted p < 0.001) (**Figure 2a**) and energy intake (AOR: 1.475; 95% CI: 1.116-1.947; adjusted p < 0.01) (**Figure 2c**). Additionally, there was a dose-dependent increment in AORs associated with high DFS as fruit and vegetable intake decreased and energy intake increased. Although SF analysis indicated the absence of a synergistic interaction between the dietary factors examined (**Supplemental Table 5 [I] and [II]**), they may act independently to influence AD presentation. In contrast, the association between DFS and AD chronicity was not statistically significant in high fruit and vegetable intake (**Figure 2b**) or low energy intake (**Figure 2d**). This suggests the potential importance of dietary modification, particularly increasing fruit and vegetable intake or reducing energy intake, in lowering the odds of AD chronicity associated with frequent high-fat food intake.

## 5.4 Association Between Frequency and Dietary Fat Intake with AD

Finally, we studied the association between dietary habits characterized by frequent intake of high-fat food diets and high total dietary fat intake with the presentation of AD. Understanding this distinction is crucial as it provides insights into how different aspects of dietary fat consumption may impact the manifestation of AD. Compared to individuals who infrequently consumed high-fat foods while maintaining a low dietary fat intake, those with increased frequency of high-fat food intake exhibited higher associated odds for AD presentation (AOR: 1.513; 95% CI: 1.219-1.882; adjusted p < 0.001). Similarly, the associated odds for AD presentation increased further with higher dietary fat intake (AOR: 1.887; 95% CI: 1.520-2.348; adjusted p < 0.001) (**Figure 3**). Notably, the influence of high-fat food intake frequency on AD odds was independent of the overall intake amount (**Supplemental Table 5 [III]**). Taken together, the key findings emphasized the importance of moderating the intake frequency of high-fat foods as potentially more crucial for lowering the odds associated with AD presentation than solely focusing on reducing overall dietary fat intake.

# 6 Discussion

Our cross-sequential study found a significant dose-dependent association between frequent high-fat food intake and the presentation and chronicity of AD, but not its severity or persistency. The association was specific to AD and did not extend to other non-allergic related secondary outcomes, such as acne, drug allergies, tooth decay, dry skin, HAV infection, or emotional discomfort. Additionally, there was a similar dose-dependent relationship between high-fat food intake and other allergic conditions, including HDM allergy, AS, and AR. Interestingly, more frequent consumption of high-fat foods, even in lower amounts, was associated with higher odds of AD presentation. These findings suggest that the frequency of high-fat food consumption, rather than solely the overall amount of dietary fat, plays a critical role in AD susceptibility, offering a potential target for dietary interventions aimed at reducing the occurrence of AD.

High-fat diets are implicated in promoting AD and allergic diseases through several common underlying mechanisms. First, excessive dietary fat can dysregulate macrophage function, increasing inflammation [43]. Second, lipid mediators, particularly saturated fatty acids like palmitic acid, activate immune cells via toll-like receptor (TLR) 2 and TLR4, triggering inflammatory responses [44-46]. Third, high-fat intake can disrupt gut microbiota, compromising gut integrity and increasing inflammatory cell infiltration [47]. Our findings support an association between high-fat food intake and AD, particularly in symptom presentation and chronicity. However, the absence of significant associations with AD severity and persistency suggests distinct underlying mechanisms. AD severity was defined in our study by late-night sleep disturbances due to itching and itch-associated neuromodulators and cytokines like interleukin-31 and thymic stromal lymphopoietin may not be influenced by high-fat intake [48, 49]. For AD persistency, the small number of recovered AD cases may have reduced statistical power, making associations harder to detect in this current study. Additionally, confounding factors such as antioxidant-rich diets or poor sleep habits could influence inflammatory pathways, modulating symptoms persistence [50, 51].

To better understand the complex role of dietary fats in the severity and persistency of AD, future studies should integrate functional assessments of microbiome composition, immune responses, and metabolomic and lipidomic profiles, alongside evaluations of sleep patterns and concurrent dietary habits. These investigations can be achieved through multi-omics approaches, incorporating stool and blood analyses to capture metabolic and immunological markers, actigraphy to monitor sleep disturbances, and detailed dietary logs or 24-hour recalls to examine overall nutrition [52]. Combining these methods will provide a holistic view of how dietary fats, lifestyle factors, and individual metabolic variations influence long-term AD progression and symptom persistence.

The retrospective nature of our study limits our ability to establish a causal relationship between the frequency of high-fat food intake and AD phenotypes. While our findings suggest an association between frequent intake of high-fat foods and AD presentation, caution is warranted in interpreting these findings. Dietary habits alone are neither exclusive nor sufficient to entirely modify AD risk, as other factors, such as sleep patterns and allergen exposures, also contribute to AD susceptibility [51, 53]. Furthermore, individual differences, cultural influences, and personal eating experiences can shape dietary habits and their impact on AD progression and management [54]. Therefore, the study’s findings should be interpreted in consideration of these potential confounding factors and future research would benefit from a more comprehensive assessment of such variables. Randomized controlled trials focusing on dietary interventions to reduce the frequency of high-fat food intake are needed to clarify the temporal sequence between dietary fats and AD. We also acknowledge the potential for recall or reporting bias in dietary data. To address this, we implemented several measures to mitigate these limitations. The direct administration of a validated FFQ by trained investigators provided participants with the opportunity to clarify any doubts during data collection. The FFQ was designed to be simple, straightforward, and cost-effective for assessing dietary habits in a large and diverse population. Moreover, the FFQ was validated for the adult population [55] with the protocol has been adjusted to include dietary questions applicable to adults, with guidelines for customizing the food list to regional dietary patterns [56]. Lastly, our study highlights the need for broader ethnic representation. Future studies should aim to replicate these findings in other ethnic groups, including Malay, Indian, and other Asian populations, to enhance the generalizability of the results. To achieve this, we plan to leverage large, independent datasets in Singapore, such as the multiethnic cohort (MEC) population [57], the Growing Up in Singapore Towards Healthy Outcomes (GUSTO) longitudinal birth cohort [58], and the Health for Life in Singapore (HELIOS) population cohort [59]. Additionally, we propose conducting randomized controlled trials to further validate the observed associations and establish causal relationships. Such efforts will provide deeper insights into how dietary habits and cultural factors uniquely influence AD across diverse ethnicities.

In conclusion, our study highlights an association between frequent intake of high-fat foods and AD, particularly in symptom presentation and chronicity. These findings emphasize the clinical importance of moderation in dietary fat intake as a potential and promising strategy for managing AD. While further research is necessary to explore underlying mechanisms and validate these results across diverse populations, our study provides a strong foundation for investigating dietary interventions aimed at reducing high-fat food intake to mitigate AD symptoms and improve AD outcomes.

# 7 Declaration

# This study was conducted in accordance with the principles of the Declaration of Helsinki and Good Clinical Practices, and in compliance with local regulatory requirements. The cross-sectional studies in Singapore were conducted on the National University of Singapore (NUS) campus annually between 2005 and 2019 with the approval of the Institutional Review Board (NUS-IRB Reference Code: NUS-07-023, NUS-09-256, NUS-10-445, NUS-13-075, NUS-14-150, and NUS-18-036) and by the Helsinki declaration. The cross-sectional studies in Malaysia were held in the Universiti Tunku Abdul Rahman (UTAR), and Sunway University. Ethical approval was granted respectively from the Scientific and Ethical Review Committee (SERC) of UTAR (Ref. code: U/SERC/03/2016) and Sunway University Research Ethics Committee (Ref. code: SUREC 2019/029). Before the data collection, all participants involved signed an informed consent form. Only participants aged 18 years old or above are allowed to participate and those under 21 years old must obtain parental/guardian consent. All identifiable information will never be used in a publication or presentation. At the earliest possible stage, all raw data have been de-identified. Data and samples collected will be studied as a whole and not individually.

# 8 Acknowledgements

The authors would like to acknowledge and sincerely thank all our participants and their family members for willing to participate in this study.

# 9 Funding Disclosure

F.T.C. received grants from the National University of Singapore (N-154-000-038-001 (E-154-00-0017-01); C141-000-077-001 (E-141-00-0096-01)), Singapore Ministry of Education Academic Research Fund (R-154-000-191-112; R-154-000-404-112; R-154-000-553-112; R-154-000-565-112; R-154-000-630-112; R-154-000-A08-592; R-154-000-A27-597; R-154-000-A91-592; R-154-000-A95-592; R154-000-B99-114), Biomedical Research Council (BMRC) (Singapore) (BMRC/01/1/21/18/077; BMRC/04/1/21/19/315; BMRC/APG2013/108), Singapore Immunology Network (SIgN-06-006; SIgN-08-020), National Medical Research Council (NMRC) (Singapore) (NMRC/1150/2008; OFIRG20nov-0033; MOH-001636 (OFLCG23may-0038, A-8002641-00-00)), National Research Foundation (NRF) (Singapore) (NRF-MP-2020-0004), Singapore Food Agency (SFA) (SFS_RND_SUFP_001_04; W22W3D0006), Singapore's Economic Development Board (EDB) (A-8002576-00-00), and the Agency for Science Technology and Research (A*STAR) (Singapore) (H17/01/a0/008; and APG2013/108). The funding agencies had no role in the study design, data collection and analysis, decision to publish, or preparation of the manuscript.

# 10 Conflict of Interests

F.T.C reports grants from the National University of Singapore, Singapore Ministry of Education Academic Research Fund, Singapore Immunology Network, National Medical Research Council (NMRC) (Singapore), Biomedical Research Council (BMRC) (Singapore), National Research Foundation (NRF) (Singapore), Singapore Food Agency (SFA), Singapore’s Economic Development Board (EDB), and the Agency for Science Technology and Research (A*STAR) (Singapore),  during the conduct of the study; and consulting fees from Sime Darby Technology Centre; First Resources Ltd; Genting Plantation, Olam International, Musim Mas, and Syngenta Crop Protection, outside the submitted work. The other authors declare no other competing interests. This research is supported by the National Research Foundation Singapore under its Open Fund-Large Collaborative Grant (MOH-001636) (A-8002641-00-00) and administered by the Singapore Ministry of Health’s National Medical Research Council. The other authors declare no other competing interests.

# 11 Authors Contributions

F.T.C.: Conceptualization and supervision of current research study. J.J.L.: Literature review, data analysis, and writing of manuscript. J.J.L., K.R., Y.H.S, and M.H.L.: Recruitment of participants and data collation. All authors read and approved the final manuscript. All authors report no conflicts of interest.

# 12 Consent for Publication

All authors have read and consented to publication of this manuscript.

# 13 Availability of Data and Materials

All data used and included in this study are available from the corresponding author (F.T.C.).

# 14 References

1. Sacotte R, Silverberg JI. Epidemiology of adult atopic dermatitis. *Clin Dermatol*. 2018;36(5):595-605. doi:10.1016/j.clindermatol.2018.05.007
2. Faye O, Flohr C, Kabashima K, et al. Atopic dermatitis: A global health perspective. *J Eur Acad Dermatol Venereol*. 2024;38(5):801-811. doi:10.1111/jdv.19723
3. Lim JJ, Lim YYE, Ng JY, et al. An update on the prevalence, chronicity, and severity of atopic dermatitis and the associated epidemiological risk factors in the Singapore/Malaysia Chinese young adult population: A detailed description of the Singapore/Malaysia Cross-Sectional Genetics Epidemiology Study (SMCGES) cohort. *World Allergy Organ J*. 2022;15(12):100722. Published 2022 Nov 18. doi:10.1016/j.waojou.2022.100722
4. Luger T, Amagai M, Dreno B, et al. Atopic dermatitis: Role of the skin barrier, environment, microbiome, and therapeutic agents. *J Dermatol Sci*. 2021;102(3):142-157. doi:10.1016/j.jdermsci.2021.04.007
5. Kantor R, Kim A, Thyssen JP, Silverberg JI. Association of atopic dermatitis with smoking: A systematic review and meta-analysis. *J Am Acad Dermatol*. 2016;75(6):1119-1125.e1. doi:10.1016/j.jaad.2016.07.017
6. Halling-Overgaard AS, Hamann CR, Holm RP, et al. Atopic dermatitis and alcohol use - a meta-analysis and systematic review. *J Eur Acad Dermatol Venereol*. 2018;32(8):1238-1245. doi:10.1111/jdv.14814
7. Silverberg NB, Lee-Wong M, Yosipovitch G. Diet and atopic dermatitis. *Cutis*. 2016;97(3):227-232.
8. Lim JJ, Lim YYE, Ng JY, et al. Burgers, Fast Foods, and Increased Associated Risk for Atopic Dermatitis: A Cross-Sectional Study of Dietary Habits among Young Chinese Adults in Singapore/Malaysia. *Dermatology*. 2024;240(1):77-84. doi:10.1159/000533942
9. Lim JJ, Reginald K, Say YH, Liu MH, Chew FT. Dietary Protein Intake and Associated Risks for Atopic Dermatitis, Intrinsic Eczema, and Allergic Sensitization among Young Chinese Adults in Singapore/Malaysia: Key Findings from a Cross-sectional Study. *JID Innov*. 2023;3(6):100224. Published 2023 Aug 21. doi:10.1016/j.xjidi.2023.100224
10. Pingali, P., 2007. Westernization of Asian diets and the transformation of food systems: Implications for research and policy. Food Policy 32, 281–298. doi:10.1016/j.foodpol.2006.08.001
11. Drewnowski A, Popkin BM. The nutrition transition: new trends in the global diet. *Nutr Rev*. 1997;55(2):31-43. doi:10.1111/j.1753-4887.1997.tb01593.x
12. Venter C, Meyer RW, Nwaru BI, et al. EAACI position paper: Influence of dietary fatty acids on asthma, food allergy, and atopic dermatitis. *Allergy*. 2019;74(8):1429-1444. doi:10.1111/all.13764
13. *Total Fat Intake for the Prevention of Unhealthy Weight Gain in Adults and Children: WHO Guideline*. Geneva: World Health Organization; 2023.
14. Ellwood P, Asher MI, García-Marcos L, et al. Do fast foods cause asthma, rhinoconjunctivitis and eczema? Global findings from the International Study of Asthma and Allergies in Childhood (ISAAC) phase three. *Thorax*. 2013;68(4):351-360. doi:10.1136/thoraxjnl-2012-202285
15. Lim JJ, Reginald K, Say YH, Liu MH, Chew FT. A dietary pattern of frequent plant-based foods intake reduced the associated risks for atopic dermatitis exacerbation: Insights from the Singapore/Malaysia cross-sectional genetics epidemiology cohort. *BMC Public Health*. 2023;23(1):1818. Published 2023 Sep 19. doi:10.1186/s12889-023-16736-y
16. Lim JJ, Lim SW, Reginald K, Say YH, Liu MH, Chew FT. Association of frequent intake of trans fatty acids and saturated fatty acids in diets with increased susceptibility of atopic dermatitis exacerbation in young Chinese adults: A cross-sectional study in Singapore/Malaysia. *Skin Health Dis*. 2024;4(4):e330. Published 2024 Jun 26. doi:10.1002/ski2.330
17. Lim JJ, Reginald K, Say YH, Liu MH, Chew FT. Frequent intake of high fiber and probiotic diets lowers risks associated with atopic dermatitis and house dust mite allergy: a cross-sequential study of young Chinese adults from Singapore and Malaysia. *Eur J Nutr*. 2024;64(1):38. Published 2024 Nov 30. doi:10.1007/s00394-024-03524-6
18. Lim JJ, Reginald K, Say YH, Liu MH, Chew FT. Associations Between Self-Reported Dietary Intake and Atopic Dermatitis Risk in Young Adults From Singapore and Malaysia. *Clin Exp Allergy*. Published online January 21, 2025. doi:10.1111/cea.14629
19. Singstat, 2020. Census of Population 2020 Statistical Release. Retrieved from https://www.singstat.gov.sg/-/media/files/publications/cop2020/sr1/findings.pdf on 2023 Feb
20. Charan J, Biswas T. How to calculate sample size for different study designs in medical research?. *Indian J Psychol Med*. 2013;35(2):121-126. doi:10.4103/0253-7176.116232
21. Jeyagurunathan A, Abdin E, Shafie S, et al. Asthma Prevalence and its Risk Factors Among a Multi-Ethnic Adult Population. *Yale J Biol Med*. 2021;94(3):417-427. Published 2021 Sep 30.
22. Wang XS, Tan TN, Shek LP, et al. The prevalence of asthma and allergies in Singapore; data from two ISAAC surveys seven years apart. *Arch Dis Child*. 2004;89(5):423-426. doi:10.1136/adc.2003.031112
23. Wong QYA, Lim JJ, Ng JY, et al. Allergic rhinitis in Chinese young adults from the Singapore/Malaysia cross-sectional genetics epidemiology study (SMCGES) cohort: Prevalence, patterns, and epidemiology of allergic rhinitis. *World Allergy Organ J*. 2022;15(10):100704. Published 2022 Oct 7. doi:10.1016/j.waojou.2022.100704
24. Wong QYA, Lim JJ, Ng JY, et al. An updated prevalence of asthma, its phenotypes, and the identification of the potential asthma risk factors among young Chinese adults recruited in Singapore. *World Allergy Organ J*. 2023;16(3):100757. Published 2023 Mar 20. doi:10.1016/j.waojou.2023.100757
25. Asher MI, Keil U, Anderson HR, Crane J, Martinez F, Mitchell EA, et al. International Study of Asthma and Allergies in Childhood (ISAAC): rationale and methods. *Eur Respir J*. 1995;8(3):483-491. doi:10.1183/09031936.95.08030483
26. Hanifin J, Rajka G. Diagnostic features of atopic eczema. *Acta Derm Venereol.* 1980;**92**:44–47. doi: 10.2340/00015555924447
27. Williams HC, Burney PG, Hay RJ, et al. The U.K. Working Party’s Diagnostic Criteria for Atopic Dermatitis. I. Derivation of a minimum set of discriminators for atopic dermatitis. *Br J Dermatol.* 1994;**131**(3):383–396. doi: 10.1111/j.1365-2133,1994.tb08530.x
28. Chew FT, Lim SH, Goh DY, Lee BW. Sensitization to local dust-mite fauna in Singapore. *Allergy.* 1999;**54**(11):1150–1159. doi: 10.1034/j.1398-9995.1999.00050.x.
29. Chew F.T., Zhang L., Ho T.M., Lee B.W. House dust mite fauna of tropical Singapore. *Clin Exp Allergy.* 1999;29:201–206.
30. Andiappan AK, Puan KJ, Lee B, Nardin A, Poidinger M, Connolly J, et al. Allergic airway diseases in a tropical urban environment are driven by dominant mono-specific sensitization against house dust mites. *Allergy*. 2014;69(4):501-509. doi:10.1111/all.12364
31. Fishbein AB, Silverberg JI, Wilson EJ, Ong PY. Update on Atopic Dermatitis: Diagnosis, Severity Assessment, and Treatment Selection. *J Allergy Clin Immunol Pract*. 2020;8(1):91-101. doi:10.1016/j.jaip.2019.06.044
32. Fasseeh AN, Elezbawy B, Korra N, et al. Burden of Atopic Dermatitis in Adults and Adolescents: a Systematic Literature Review. *Dermatol Ther (Heidelb)*. 2022;12(12):2653-2668. doi:10.1007/s13555-022-00819-6
33. Ramirez FD, Chen S, Langan SM, et al. Association of Atopic Dermatitis With Sleep Quality in Children. *JAMA Pediatr*. 2019;173(5):e190025. doi:10.1001/jamapediatrics.2019.0025
34. Say YH, Sio YY, Heng AHS, et al. Golgin A7 family member B (GOLGA7B) is a plausible novel gene associating high glycaemic index diet with acne vulgaris. *Exp Dermatol*. 2022;31(8):1208-1219. doi:10.1111/exd.14575
35. Ellwood P, Asher MI, Björkstén B, Burr M, Pearce N, Robertson CF. Diet and asthma, allergic rhinoconjunctivitis and atopic eczema symptom prevalence: an ecological analysis of the International Study of Asthma and Allergies in Childhood (ISAAC) data. ISAAC Phase One Study Group. Eur Respir J. 2001;17(3):436–443. 10.1183/09031936.01.17304360
36. Health Promotion Board, 2023. Energy and Nutrient Composition of Food. Retrieved from https://focos.hpb.gov.sg/eservices/ENCF/ on 2023 Jul
37. Manousos O, Day NE, Trichopoulos D, Gerovassilis F, Tzonou A, Polychronopoulou A. Diet and colorectal cancer: a case-control study in Greece. *Int J Cancer*. 1983;32(1):1-5. doi:10.1002/ijc.2910320102
38. Lim JJ, Reginald K, Say YH, Liu MH, Chew FT. A Dietary Pattern for High Estimated Total Fat Amount Is Associated with Enhanced Allergy Sensitization and Atopic Diseases among Singapore/Malaysia Young Chinese Adults. *Int Arch Allergy Immunol*. 2023;184(10):975-984. doi:10.1159/000530948
39. Cho SI, Lee H, Lee DH, Kim KH. Association of frequent intake of fast foods, energy drinks, or convenience food with atopic dermatitis in adolescents. *Eur J Nutr*. 2020;59(7):3171-3182. doi:10.1007/s00394-019-02157-4
40. M. Cortina-Borja, A. D. Smith, O. Combarros, D. J. Lehmann, The synergy factor: A statistic to measure interactions in complex diseases. *BMC Research Notes*. 2 (2009), doi:10.1186/1756-0500-2-105.
41. Colquhoun D. An investigation of the false discovery rate and the misinterpretation of p-values. *R Soc Open Sci*. 2014;1(3):140216. Published 2014 Nov 19. doi:10.1098/rsos.140216
42. Hersoug LG, Linneberg A. The link between the epidemics of obesity and allergic diseases: does obesity induce decreased immune tolerance?. *Allergy*. 2007;62(10):1205-1213. doi:10.1111/j.1398-9995.2007.01506.x
43. Kiran S, Rakib A, Kodidela S, Kumar S, Singh UP. High-Fat Diet-Induced Dysregulation of Immune Cells Correlates with Macrophage Phenotypes and Chronic Inflammation in Adipose Tissue. *Cells*. 2022;11(8):1327. Published 2022 Apr 13. doi:10.3390/cells11081327
44. Snodgrass RG, Huang S, Choi IW, Rutledge JC, Hwang DH. Inflammasome-mediated secretion of IL-1β in human monocytes through TLR2 activation; modulation by dietary fatty acids. *J Immunol*. 2013;191(8):4337-4347. doi:10.4049/jimmunol.1300298
45. Rocha DM, Caldas AP, Oliveira LL, Bressan J, Hermsdorff HH. Saturated fatty acids trigger TLR4-mediated inflammatory response. *Atherosclerosis*. 2016;244:211-215. doi:10.1016/j.atherosclerosis.2015.11.015
46. Korbecki J, Bajdak-Rusinek K. The effect of palmitic acid on inflammatory response in macrophages: an overview of molecular mechanisms. *Inflamm Res*. 2019;68(11):915-932. doi:10.1007/s00011-019-01273-5
47. Hamamah S, Amin A, Al-Kassir AL, Chuang J, Covasa M. Dietary Fat Modulation of Gut Microbiota and Impact on Regulatory Pathways Controlling Food Intake. *Nutrients*. 2023;15(15):3365. Published 2023 Jul 28. doi:10.3390/nu15153365
48. Labib A, Vander Does A, Yosipovitch G. Nemolizumab for atopic dermatitis. *Drugs Today (Barc)*. 2022;58(4):159-173. doi:10.1358/dot.2022.58.4.3378056
49. Kwatra SG, Misery L, Clibborn C, Steinhoff M. Molecular and cellular mechanisms of itch and pain in atopic dermatitis and implications for novel therapeutics. *Clin Transl Immunology*. 2022;11(5):e1390. Published 2022 May 9. doi:10.1002/cti2.1390
50. Wang S, Dan W, Wang Z, Sun Y, Zhang G. Causal relationships between dietary antioxidant vitamin intake and atopic dermatitis: A two-sample Mendelian randomization study. *Skin Res Technol*. 2024;30(8):e13883. doi:10.1111/srt.13883
51. Chang YS, Chiang BL. Sleep disorders and atopic dermatitis: A 2-way street?. *J Allergy Clin Immunol*. 2018;142(4):1033-1040. doi:10.1016/j.jaci.2018.08.005
52. Wastyk HC, Fragiadakis GK, Perelman D, et al. Gut-microbiota-targeted diets modulate human immune status. *Cell*. 2021;184(16):4137-4153.e14. doi:10.1016/j.cell.2021.06.019
53. Kantor R, Silverberg JI. Environmental risk factors and their role in the management of atopic dermatitis. *Expert Rev Clin Immunol*. 2017;13(1):15-26. doi:10.1080/1744666X.2016.1212660
54. Shepherd R. Social determinants of food choice.*Proc Nutr Soc*. 1999;58(4):807-812. doi:10.1017/s0029665199001093
55. Ellwood P, Asher MI, Billo NE, et al. The Global Asthma Network rationale and methods for Phase I global surveillance: prevalence, severity, management and risk factors. *Eur Respir J*. 2017;49(1):1601605. Published 2017 Jan 11. doi:10.1183/13993003.01605-2016
56. Global Asthma Network . 2016. The global asthma Network manual for global Surveillance: prevalence, severity, Management and risk factors: Global Asthma Network.
57. Whitton C, Ho JCY, Tay Z, et al. Relative Validity and Reproducibility of a Food Frequency Questionnaire for Assessing Dietary Intakes in a Multi-Ethnic Asian Population Using 24-h Dietary Recalls and Biomarkers. *Nutrients*. 2017;9(10):1059. Published 2017 Sep 25. doi:10.3390/nu9101059
58. Soh SE, Chong YS, Kwek K, et al. Insights from the Growing Up in Singapore Towards Healthy Outcomes (GUSTO) cohort study. *Ann Nutr Metab*. 2014;64(3-4):218-225. doi:10.1159/000365023
59. Lam CCB, Mina T, Xie W, et al. The relationships between sleep and adiposity amongst multi-ethnic Asian populations: a cross-sectional analysis of the Health for Life in Singapore (HELIOS) study. *Int J Obes (Lond)*. Published online November 19, 2024. doi:10.1038/s41366-024-01666-5

# 15 Tables

**Table 1.** An overview of the demographic distribution of 13,561 young Chinese adults from the Singapore/Malaysia Cross-sectional Genetics Epidemiology Study (SMCGES) cohort. Participants were categorized based on their dietary fat scores using the frequency-based dietary index, Diet Quality based on Dietary Fat Score (DQDFS).

| **Variables** | **Diet Quality based on Dietary Fat Score (DQDFS)^1^** | | |  | **Chi-square p-value^2^** | | |
| --- | --- | --- | --- | --- | --- | --- | --- |
|  | **Low DFS**  **(*n* = 4343)** | **Moderate DFS**  ***(n* = 3984)** | **High DFS**  **(*n* = 4658)** | **Overall Population**  **(n = 13,561)** | **Low DFS vs. Moderate DFS** | **Moderate DFS vs. High DFS** | **Low DFS vs. High DFS** |
| **Mean Age (± SD)** | 22.91 ± 6.58 | 22.52 ± 5.89 | 22.11 ± 5.09 | 22.51 ± 5.90 | - | - | - |
| **Sex** | | | | | | | |
| - Female | 2829 (65.1%) | 2345 (58.9%) | 2526 (54.2%) | 8060 (59.4%) | **<0.0001** | **<0.0001** | **<0.0001** |
| - Male | 1514 (34.9%) | 1639 (41.1%) | 2132 (45.8%) | 5501 (40.6%) |  |  |  |
| **Body Mass Index, Asian Class (kg/m^2^) (*n*, %)** | | | | | | | |
| - Healthy - (18.0-23.0) | 2402 (55.3%) | 2223 (55.8%) | 2519 (54.1%) | 7445 (54.9%) | 0.153 (ns) | 0.116 (ns) | **0.001** |
| - Underweight (<18.0) | 785 (18.1%) | 668 (16.8%) | 770 (16.5%) | 2327 (17.2%) |  |  |  |
| - Overweight (>23.0) | 615 (14.2%) | 607 (15.2%) | 781 (16.8%) | 2092 (15.4%) |  |  |  |
| NA | 541 (12.5%) | 486 (12.2%) | 588 (12.6%) | 1697 | - | - | - |
| **Parental Eczema (*n*, %)** | | | | | | | |
| - No | 3744 (86.2%) | 3507 (88.0%) | 4082 (87.6%) | 11,831 (87.2%) | **<0.05** | 0.343 (ns) | 0.142 (ns) |
| - Yes | 538 (12.4%) | 429 (10.8%) | 533 (11.4%) | 1558 (11.5%) |  |  |  |
| NA | 61 (1.4%) | 48 (1.2%) | 43 (0.9%) | 172 | - | - | - |
| **Tobacco Smoking Status (*n*, %)** | | | | | | | |
| - Non-smoker | 3982 (91.7%) | 3663 (91.9%) | 4258 (91.4%) | 12,395 (91.4%) | **<0.05** | **<0.05** | **<0.0001** |
| - Ex-smoker | 23 (0.5%) | 42 (1.1%) | 63 (1.4%) | 135 (1.00%) |  |  |  |
| - Smoker | 50 (1.2%) | 44 (1.1%) | 81 (1.7%) | 183 (1.35%) |  |  |  |
| NA | 288 (6.6%) | 235 (5.9%) | 256 (5.5%) | 848 | - | - | - |
| **Use of Alcohol (*n*, %)** | | | | | | | |
| - Non-drinker | 2114 (48.7%) | 1688 (42.4%) | 2499 (53.6%) | 5830 (43.0%) | **<0.0001** | **<0.0001** | **<0.0001** |
| - Occasionally | 1897 (43.7%) | 1990 (49.9%) | 1785 (38.3%) | 6643 (49.0%) |  |  |  |
| - Frequent | 58 (1.3%) | 77 (1.9%) | 132 (2.8%) | 277 (2.04%) |  |  |  |
| NA | 274 (6.3%) | 229 (5.7%) | 242 (5.2%) | 811 | - | - | - |

^1^ A total of 576 participants were excluded because they did not fit into any of the DQDFS categories.

^2^ A chi-square *p-*value < 0.05 was adjusted by False Discovery Rate (FDR) for multiple comparisons. Statistically significant p-values were bolded.

**Table 2.** Association between the intake frequency of high-fat foods, as assessed by the frequency-based dietary index – Diet Quality based on Dietary Fat Score (DQDFS), and various outcomes among 13,561 young Chinese adults from the Singapore/Malaysia Cross-sectional Genetics Epidemiology Study (SMCGES) cohort.

| Outcome | **AOR** | **95% CI** | **p** |
| --- | --- | --- | --- |
| I. **Ever Atopic Dermatitis (AD) (Non-allergic non-eczema vs. AD cases)** | | | |
| Low Dietary Fat Score | 1.000 | REF | - |
| Moderate Dietary Fat Score | 1.245 | 1.068-1.452 | **0.005** |
| High Dietary Fat Score | 1.554 | 1.340-1.802 | **<0.001** |
| II. **Ever Allergic Rhinitis (AR) (Non-allergic non-rhinitis vs. AR cases)** | | | |
| Low Dietary Fat Score | 1.000 | REF | - |
| Moderate Dietary Fat Score | 1.214 | 1.051-1.403 | **0.008** |
| High Dietary Fat Score | 1.255 | 1.092-1.442 | **0.001** |
| III. **Ever Allergic Asthma (AS) (Non-allergic non-asthma vs. AS cases)** | | | |
| Low Dietary Fat Score | 1.000 | REF | - |
| Moderate Dietary Fat Score | 1.187 | 1.006-1.400 | 0.043 |
| High Dietary Fat Score | 1.279 | 1.089-1.503 | **0.003** |
| IV. **House Dust Mites (HDMs) Allergy (Skin prick test [SPT] negative individuals vs. SPT positive individuals)** | | | |
| Low Dietary Fat Score | 1.000 | REF | - |
| Moderate Dietary Fat Score | 1.175 | 1.062-1.300 | **0.002** |
| High Dietary Fat Score | 1.376 | 1.246-1.519 | **<0.001** |
| V. **Acne Vulgaris (non-acne vs. acne cases)** | | | |
| Low Dietary Fat Score | 1.000 | REF | - |
| Moderate Dietary Fat Score | 1.081 | 0.946-1.235 | 0.252 |
| High Dietary Fat Score | 0.978 | 0.961-1.111 | 0.731 |
| VI. **Dry Skin (non-dry skin vs. dry skin cases)** | | | |
| Low Dietary Fat Score | 1.000 | REF | - |
| Moderate Dietary Fat Score | 1.206 | 1.021-1.425 | 0.027 |
| High Dietary Fat Score | 1.169 | 0.998-1.371 | 0.053 |
| VII. **Chronic Rhinosinusitis (non-chronic rhinosinusitis vs. chronic rhinosinusitis cases)** | | | |
| Low Dietary Fat Score | 1.000 | REF | - |
| Moderate Dietary Fat Score | 1.141 | 0.917-1.420 | 0.237 |
| High Dietary Fat Score | 1.072 | 0.869-1.321 | 0.517 |
| VIII. **Tooth Decay (no tooth decay vs. tooth decay)** | | | |
| Low Dietary Fat Score | 1.000 | REF | - |
| Moderate Dietary Fat Score | 1.093 | 0.811-1.475 | 0.559 |
| High Dietary Fat Score | 0.891 | 0.669-1.187 | 0.432 |
| IX. **Hepatitis A Virus (HAV) Infection (non-infected vs. infected cases)** | | | |
| Low Dietary Fat Score | 1.000 | REF | - |
| Moderate Dietary Fat Score | 0.666 | 0.136-2.727 | 0.580 |
| High Dietary Fat Score | 1.020 | 0.279-3.732 | 0.975 |
| X. **Emotional Discomfort (control vs. cases)** | | | |
| Low Dietary Fat Score | 1.000 | REF | - |
| Moderate Dietary Fat Score | 1.012 | 0.740-1.385 | 0.941 |
| High Dietary Fat Score | 1.056 | 0.783-1.427 | 0.720 |
| XI. **Drug Allergy (no drug allergy vs. drug allergy)** | | | |
| Low Dietary Fat Score | 1.000 | REF | - |
| Moderate Dietary Fat Score | 0.985 | 0.856-1.135 | 0.838 |
| High Dietary Fat Score | 0.921 | 0.802-1.057 | 0.240 |
| XII. **Pain Medication Allergy (no pain drug allergy vs. pain drug allergy)** | | | |
| Low Dietary Fat Score | 1.000 | REF | - |
| Moderate Dietary Fat Score | 1.298 | 0.721-2.347 | 0.383 |
| High Dietary Fat Score | 1.124 | 0.626-2.032 | 0.695 |

Results are from multivariable logistic regression analysis on the variables associated with various outcomes as indicated. Values are presented as adjusted odds ratio (AOR), 95% confidence intervals (CI), and *p*-value. P-value was adjusted by False Discovery Rate (FDR) for multiple comparisons and p-value < 0.05 was statistically significant and written in bold. P-values > 0.05 was not statistically significant (ns). Multivariable analysis was adjusted for age, sex, body mass index, parental eczema, tobacco smoking, and use of alcohol
